# Eligibility of real-world patients for aspirin primary prevention trials in cardiovascular disease

**DOI:** 10.1186/s12916-026-04654-w

**Published:** 2026-01-27

**Authors:** Michael Holder, Daniel R. Morales, Peter Hanlon, David A McAllister, Bruce Guthrie

**Affiliations:** 1 Advanced Care Research Centre, Usher Institute, University of Edinburgh, Edinburgh, EH16 4UX UK; 2 Division of Population Health and Genomics, University of Dundee, Dundee, UK; 3 General Practice and Primary Care, School of Health and Wellbeing, University of Glasgow, Glasgow, UK; 4 Health Economics and Health Technology Assessment, School of Health and Wellbeing, University of Glasgow, Glasgow, UK

**Keywords:** Aspirin, Cardiovascular diseases, Primary prevention, Atherosclerosis, Randomised controlled trials as topic, External validity, Patient selection

## Abstract

**Background:**

Evidence for the net benefit of aspirin for primary prevention of cardiovascular disease (CVD) is finely balanced, leading to variation in guideline recommendations internationally. External validity of randomised clinical trial (RCT) evidence may therefore be of particular importance. The aim of this study is to characterise real-world patients according to their eligibility for guideline-cited aspirin RCTs for primary CVD prevention.

**Methods:**

Eligibility criteria from 14 RCTs were applied to a linked primary care/hospital discharge dataset of people ≥ 40 years without CVD. Proportions eligible for each trial were calculated, and characteristics of eligible and ineligible patients compared for each trial, including Cox regression analysis of event rates for major adverse cardiovascular events (MACE), major bleeding events, and non-cardiovascular mortality.

**Results:**

Of 570,211 included patients (300,500 [52.7%] women, 336,877 [59%] < 60 years), the median proportion ineligible for 14 RCTs was 90.7% (range 42.5–99.4%) and 24.0% of patients were ineligible for all RCTs. On average, trial-ineligible populations were younger (median age trial-ineligible 57.8 vs trial-eligible 62.6 years, *p* = 0.008) and a lower proportion had hypertension (23.9% vs 50.9%, *p* = 0.004), diabetes (6.4% vs 11.5%, *p* = 0.015), or a regular statin prescription (11.8% vs 26.7%, *p* = 0.001). Trial-ineligible populations had a higher hazard of MACE compared to trial-eligible in four RCTs and lower in ten (hazard ratio [HR] range across all RCTs 0.45 [95%CI 0.40–0.51] to 2.78 [95%CI 2.61–2.96]). Hazards of bleeding events in the trial-ineligible were lower than the trial-eligible in eight RCTs and higher in four (HR range across all RCTs 0.63 [95%CI, 0.59–0.66] to 1.69 [95%CI, 1.53–1.86]), and time-varying hazards of non-CVD death were consistently lower in four RCTs and higher in five (HR range across all RCTs and time points 0.29 [95%CI 0.24–0.36] to 11.42 [95%CI 9.91–13.17]).

**Conclusions:**

Compared with trial-ineligible populations within the same age and sex strata, RCTs recruited people of varying CVD risk but often excluded people at high risk of bleeding or non-CVD death, highlighting that many trials may overestimate the net benefit of aspirin for primary prevention.

**Supplementary Information:**

The online version contains supplementary material available at 10.1186/s12916-026-04654-w.

## Background

High-quality randomised clinical trials (RCTs) provide gold standard evidence to underpin clinical guideline recommendations, due to their high internal validity [1]. However, the majority of RCTs of pharmacological interventions for physical health conditions exclude the majority of people with the target condition [2], and unrepresentativeness in trial participants can compromise the real or perceived applicability or generalisability of clinical trial evidence.

Despite reductions in cardiovascular disease (CVD) incidence and mortality in recent decades, CVD remains the most common cause of death internationally [3]. Lifestyle modifications and medications for primary prevention are recommended by guidelines, including smoking cessation, weight loss, and use of statins and antihypertensives [4]. The use of aspirin for secondary prevention (preventing worsening or recurrence) of CVD is widely accepted [5], but the benefit of aspirin in primary prevention (preventing incident CVD) is less clear, with any CVD benefits finely balanced against increased risk of bleeding events [6, 7]. When benefit and harm are finely balanced, the external validity of trial evidence to people in routine care becomes more important [8], since trial-ineligible patients may be at higher risk of treatment harms than trial-eligible [2].

In 2022, the United States Preventative Services Task Force (USPSTF) advised that aspirin should be considered for the primary prevention of CVD in people aged 40–59 years with a 10-year predicted CVD risk of ≥ 10% [9]. It also recommended against aspirin use for primary prevention in adults 60 years or older, lowering the age thresholds from 50 to 69 years in the 2016 guidelines [10]. The updated recommendation was based on a new systematic review [11], which included three new RCTs published in 2018 [7, 12, 13]. Current European guidance is to avoid routine use of aspirin for patients without CVD, but it may be considered in patients whose CVD risk is exceptionally high [4], while current UK guidelines unequivocally advise against the routine use of aspirin for primary prevention of CVD [14, 15]. Aspirin for primary CVD prevention serves as a noteworthy example of how guideline developers’ interpretations of the evidence base vary both over time and geographically (Table 1) [16].

**Table 1:** Summary of international guidelines for aspirin for the primary prevention of cardiovascular disease

Organisation	Recommendation
US guidelines	
USPSTF 2022 [9]	• The decision to initiate low-dose aspirin use for the primary prevention of CVD in adults aged 40 to 59 years who have a 10% or greater 10-year CVD risk should be an individual one. Evidence indicates that the net benefit of aspirin use in this group is small. Persons who are not at increased risk for bleeding and are willing to take low-dose aspirin daily are more likely to benefit • The USPSTF recommends against initiating low-dose aspirin use for the primary prevention of CVD in adults 60 years or older
ACC/AHA 2019 [17]	• Low-dose aspirin (75–100 mg orally daily) might be considered for the primary prevention of ASCVD among select adults 40 to 70 years of age who are at higher ASCVD risk but not at increased bleeding risk • Low-dose aspirin (75–100 mg orally daily) should not be administered on a routine basis for the primary prevention of ASCVD among adults > 70 years of age • Low-dose aspirin (75–100 mg orally daily) should not be administered for the primary prevention of ASCVD among adults of any age who are at increased risk of bleeding
ADA 2019 [18]	• Aspirin therapy (75–162 mg/day) may be considered as a primary prevention strategy in those with diabetes who are at increased cardiovascular risk, after a comprehensive discussion with the patient on the benefits versus the comparable increased risk of bleeding
USPSTF 2016 [10]	• The USPSTF recommends initiating low-dose aspirin use for the primary prevention of cardiovascular disease (CVD) and colorectal cancer (CRC) in adults aged 50 to 59 years who have a 10% or greater 10-year CVD risk, are not at increased risk for bleeding, have a life expectancy of at least 10 years, and are willing to take low-dose aspirin daily for at least 10 years • The decision to initiate low-dose aspirin use for the primary prevention of CVD and CRC in adults aged 60 to 69 years who have a 10% or greater 10-year CVD risk should be an individual one. Persons who are not at increased risk for bleeding, have a life expectancy of at least 10 years, and are willing to take low-dose aspirin daily for at least 10 years are more likely to benefit. Persons who place a higher value on the potential benefits than the potential harms may choose to initiate low-dose aspirin • The current evidence is insufficient to assess the balance of benefits and harms of initiating aspirin use for the primary prevention of CVD and CRC in adults younger than 50 years • The current evidence is insufficient to assess the balance of benefits and harms of initiating aspirin use for the primary prevention of CVD and CRC in adults aged 70 years or older
International guidelines	
NICE 2023 [14]	• Do not routinely offer aspirin for primary prevention of CVD
ESC 2021 [4]	• In patients with DM at high or very high CVD risk, low-dose aspirin may be considered for primary prevention in the absence of clear contraindications • Antiplatelet therapy is not recommended in individuals with low/moderate CV risk due to the increased risk of major bleeding
SIGN 2017 [15]	• Aspirin is not recommended for primary prevention of cardiovascular disease • Aspirin is not routinely recommended in people with diabetes who do not have a diagnosis of cardiovascular disease • Aspirin is not recommended for primary prevention of cardiovascular disease in patients with hypertension

*ACC* American College of Cardiology, *ADA* American Diabetes Association, *AHA* American Heart Association, *ASCVD* atherosclerotic cardiovascular disease, *CRC* colorectal cancer, *CVD* cardiovascular disease, *DM* diabetes mellitus, *ESC* European Society of Cardiology, *NICE* National Institute of Clinical Excellence, *SIGN* Scottish Intercollegiate Guidelines Network, *USPSTF* United States Preventive Services Task Force

Given finely balanced evidence, it is important to better understand how external validity considerations might inform interpretation of internally valid RCT evidence, specifically in terms of whether baseline risk (and therefore the expected absolute benefit and harm) differs between trial-ineligible and trial-eligible patients. The aim of this study was therefore to examine the differences between people who would have been eligible and ineligible for aspirin primary CVD prevention trials.

## Methods

**Fig. 1:**
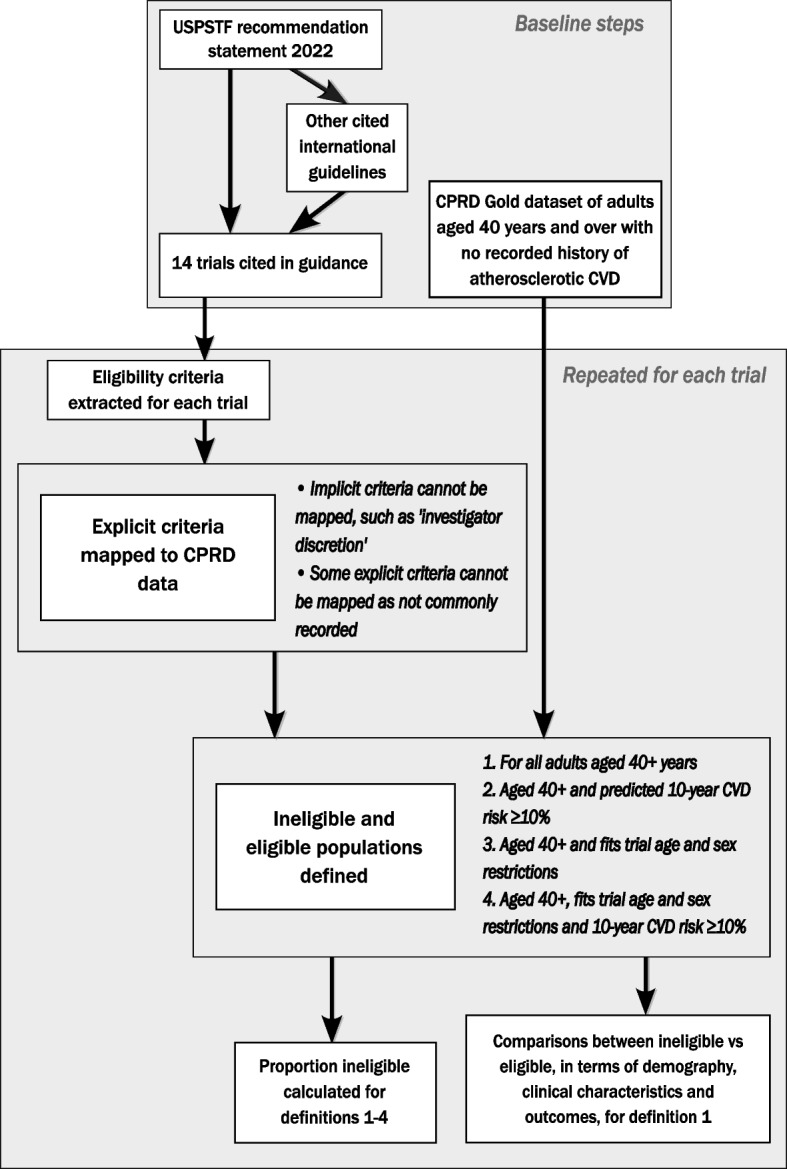
Flow diagram illustrating study methodology. Items in the top box were executed once for the study, with items within the bottom box repeated for each trial

This was a retrospective cohort study of linked United Kingdom (UK) population data, examining the characteristics and outcomes of people aged 40 years and over without CVD who were either eligible or ineligible for trials of aspirin for the primary prevention of CVD. Figure 1 provides a visual overview of the study methodology.

### Data source

Linked data were extracted from the Clinical Practice Research Datalink (CPRD) Gold dataset, which is broadly representative of the English population [19, 20]. In England, general practitioner (GP) registration is required to access National Health Service (NHS) care. The GP electronic health record is comprehensive because all specialists write to the GP at every consultation and almost all community prescribing is done by the GP, including drugs recommended by specialists. Patients can only be registered with one practice at a time and records transfer when patients move practice. CPRD Gold links data from general practice electronic health records to NHS England Hospital Episode Statistics (HES) (national hospital admission/discharge records), and Office for National Statistics (ONS) national mortality registration for three-quarters of English practices [19]. Our analysis only included practices with linked HES and ONS data, which excluded ~ 25% of English practices and all practices in other UK countries contributing GP data to CPRD Gold. Linkage between primary care and other datasets was done by NHS England using deterministic matching based on unique identifiers, with probabilistic matching where deterministic linkage is not possible [21]. There is no mechanism for obtaining data from practices who are not participating in CPRD and no ethical way to obtain data about patients who have opted out of data sharing for research.

### Study population

The study population included all adults aged 40 years and over on 30th November 2015 without a history of atherosclerotic CVD. Atherosclerotic CVD was defined as a record before baseline of myocardial infarction (MI), acute coronary syndrome, angina pectoris, peripheral arterial disease (PAD), ischaemic stroke, or transient ischaemic attack (TIA). These conditions represent all conditions where aspirin is currently recommended for secondary prevention, and the lower age limit of 40 years was chosen to reflect current guidance for assessing and managing CVD risk [14]. No additional criteria were applied, ensuring that the denominator population included the entire primary prevention group for whom CVD risk should be routinely calculated. Baseline characteristics examined included age, sex, body mass index (BMI), a range of morbidities (including hypertension, diabetes, and heart failure), and regular prescriptions (including antihypertensives, statins, and non-steroidal anti-inflammatory drugs), defined as medications prescribed at least once in each of the two 84-day periods within the 168 days before the index date. For each patient, a Charlson Comorbidity Index score [22, 23] and an electronic Frailty Index score [24] were calculated, and for all patients below the age of 85, a QRISK3 score was also calculated [25].

Outcomes examined over 3 years of follow-up were:

- Major adverse cardiovascular events (MACE), defined as cardiovascular death or hospital admission with ischaemic heart disease (including MI, unstable angina, stable angina, or coronary heart disease not otherwise specified), ischaemic stroke (or stroke not otherwise specified), TIA, or PAD;
- Major bleeding events, defined as hospital admission with a gastrointestinal or cerebrovascular bleed; and
- Non-CVD mortality, defined as all deaths not captured in our definition of MACE.

Phenotypes for all read codes and ICD-10 codes for conditions used in this study are those defined by Kuan et al. [26].

### Identification of RCTs and eligibility criteria

From six international guidelines for using aspirin for primary prevention of CVD, including USPTF [4, 9, 14, 15, 17, 18] (Table 1), 14 RCTs were identified (Additional file 1: Table S1) [6, 7, 12, 13, 27–36]. Inclusion and exclusion (eligibility) criteria were identified for each trial from trial registrations, published protocols, and published trial results, since criteria are often incompletely reported within a single source [37]. Where sources listed different criteria, we assumed that all criteria listed in any sources had been applied. In cases of direct contradiction (e.g. variations in published age ranges), the most recent source was used. Criteria were then mapped to the available routine data where possible. Frequently, implicit criteria could not be mapped, for example, ‘exclusion at investigator’s discretion’. The same was true for some explicit criteria, for example, low ankle-brachial blood pressure index (ABPI) was a key inclusion criterion in two trials (Aspirin for Asymptomatic Atherosclerosis [AAA] and Prevention Of Progression of Arterial Disease And Diabetes [POPADAD]), but was not available in the routine data, meaning that ineligibility is very likely underestimated (Additional file 1: Tables S2–S15) [6, 7, 12, 13, 27–36, 38–57]. Mapped eligibility criteria for individual RCTs were then applied to the target population. Individuals who did not meet all inclusion criteria or met any exclusion criteria were deemed ineligible; otherwise, patients were deemed eligible.

### Analysis

The proportion of the target population ineligible for each trial was calculated. Proportions eligible were also analysed in three further sub-groups: individuals with a QRISK3-predicted 10-year risk of CVD ≥ 10% (or aged 85 years or older); individuals with the same age and sex criteria as the trial; and a group meeting both age-sex criteria and with a QRISK3 score ≥ 10%. Baseline characteristics, morbidities, and selected drug exposure in trial-ineligible patients were compared to trial-eligible using Wilcoxon rank sum test for continuous variables and Pearson χ^2^ test for categorical variables. Incidence rates of MACE, major bleeding events, and non-CVD deaths were calculated and compared between trial-ineligible and trial-eligible using Cox regression to estimate hazard ratios (HRs) in the whole cohort and in subpopulations selected from patients who met each trial’s age-sex inclusion criteria. For two trials (A Study of Cardiovascular Events iN Diabetes [ASCEND] and POPADAD), whole-cohort and trial age-sex criteria population analyses were the same, as both trials enrolled men and women aged 40 years or older. We evaluated the proportional hazards assumption for all Cox models, comprising two models (whole-cohort and trial-specific age-sex criteria populations) for each of 14 trials and each of three outcomes, using the Grambsch-Therneau test and examination of Schoenfeld residuals. For MACE and bleeding events, there was some evidence of violation of proportional hazards for three whole-cohort models and five trial-specific age-sex criteria models (*p* values < 0.05 but > 0.01) but examination of Schoenfeld residuals indicated only a small variation over time. We therefore present single estimates of whole-cohort and trial-specific age-sex criteria population HRs for all MACE and bleeding event models. However, there was strong evidence of non-proportional hazards of non-cardiovascular death in both analyses. Time-varying HRs were therefore estimated for all non-cardiovascular death models. All analyses were undertaken using R version 4.1.3 (R Foundation for Statistical Computing, Vienna).

## Results

The lower age limit for trial inclusion ranged between 18 and 65 years (median 45 years), with 12 trials having a lower age limit of ≥ 40 years. Two trials (Early Treatment Diabetic Retinopathy Study [ETDRS] and Japanese Primary prevention of Atherosclerosis with aspirin for Diabetes [JPAD]) had lower age limits < 40 years, but neither included many patients younger than 40 years. Eight trials had an upper age limit (median 79.5 years, range 69–85). Four trials only included people at higher risk of CVD while four trials only included people with diabetes. All 14 trials had exclusions related to bleeding risk (such as history of bleeding, co-morbidities like peptic ulcer, or co-prescribing of other drugs causing bleeding). One trial excluded people with diabetes, six trials excluded people with chronic kidney disease and with chronic liver disease. People with any history of cancer were excluded from three trials (Additional file 1: Tables S3, S6, and S8) [28, 30, 33], and seven trials excluded people with life-limiting illnesses (Additional file 1: Tables S4–S6, S7, S9, and S14–S15) [7, 12, 29–32, 34].

The whole cohort consisted of 570,211 people aged 40 years and over without recorded CVD. Of these, 52.7% were women and most were aged 40–49 (30.3%) or 50–59 years (28.7%) (Table 2). Most people had a Charlson Comorbidity Index (CCI) of zero (60.7%) and 71.7% were categorised as ‘Fit’ using the electronic Frailty Index (eFI) [24].

**Table 2:** Demographics of participants

Characteristic	No. (%) ***N*** = 570,211
Age group (years)	
40–49	173,021 (30.3%)
50–59	163,856 (28.7%)
60–69	122,281 (21.4%)
70 +	111,053 (19.5%)
Women	300,500 (52.7%)
Minority ethnic group	28,605 (5.0%)
BMI (median (IQR)) kg/m^2^	26.6 (23.7, 30.3)
IMD quintile	
Q1 (least deprived)	158,133 (27.7%)
Q2	123,371 (21.6%)
Q3	116,646 (20.5%)
Q4	96,611 (16.9%)
Q5 (most deprived)	75,450 (13.2%)
Ever smoked	203,990 (35.8%)
Care home resident	2532 (0.4%)
Charlson score ≥ 1	223,955 (39.3%)
Moderate/severe frailty	41,713 (7.3%)
Morbidities	
Hypertension	147,778 (25.9%)
Diabetes	41,544 (7.3%)
Heart failure	5169 (0.9%)
Chronic liver disease	4918 (0.9%)
Chronic renal disease	4669 (0.8%)
Cancer	55,387 (9.7%)
Alcohol and substance misuse	26,969 (4.7%)
Regularly prescribed	
Aspirin	14,718 (2.6%)
Antihypertensives	128,569 (22.5%)
Statins	76,024 (13.3%)
PPIs	64,800 (11.4%)
NSAIDs	17,141 (3.0%)
SSRIs	35,287 (6.2%)
Number of trials ineligible for	
All 14	137,060 (24.0%)
13	112,537 (19.7%)
12	121,030 (21.2%)
11	86,142 (15.1%)
10 or fewer	113,442 (19.9%)

*BMI* body mass index, *IMD* index of multiple deprivation, *IQR* interquartile range, *PPI* proton pump inhibitor, *NSAID* non-steroidal anti-inflammatory drug, *SSRI* selective serotonin reuptake inhibitor

**Fig. 2:**
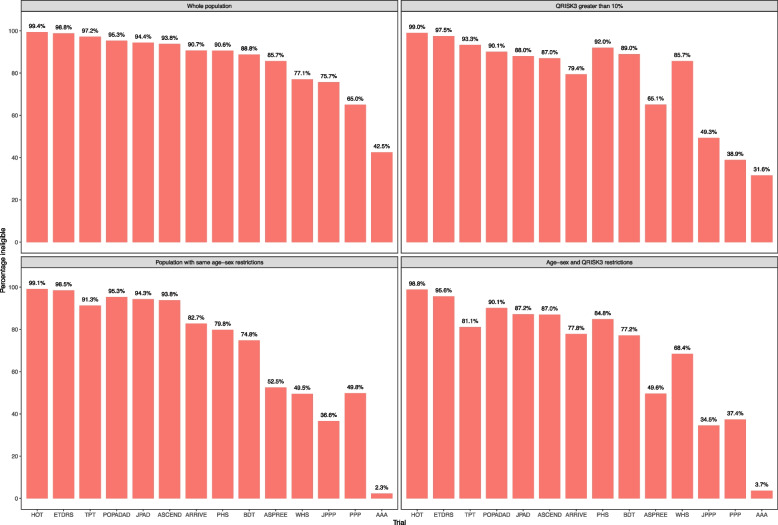
Proportions ineligible for each of the 14 trials. Trials ordered in descending order of proportion ineligible. For AAA, a key explicit criterion (an ABPI of ≤ 0.95 in at least one limb) which would exclude a large proportion of patients could not be mapped because it is very rarely recorded in routine data

The percentage of the whole cohort aged 40 years or over without CVD who were ineligible for each trial ranged from 42.5% in the AAA trial to 99.4% in the Hypertension Optimisation Trial (HOT) (median 90.7% ineligible, IQR 77.1–95.3%) (Fig. 2). In adults aged 40 years and over with a QRISK3 score of ≥ 10%, the proportion ineligible for each trial was generally slightly lower (median 87.5% ineligible, IQR 65.1–92.0%), with three trials showing a reduction of more than 20 percentage points and three trials showing an increase. In the trial-specific age-sex criteria populations, the proportion ineligible for each trial (i.e. people excluded for reasons other than age or sex) was somewhat lower (median 81.3%, IQR 49.8–94.3%), and in the population with both QRISK3 score ≥ 10% and matching individual trial age/sex criteria, lower still (median 79.5% ineligible, IQR 49.6–87.2%). Twenty-four percent of the whole cohort aged 40 years or older would have been ineligible for all 14 trials (Table 2).

**Fig. 3:**
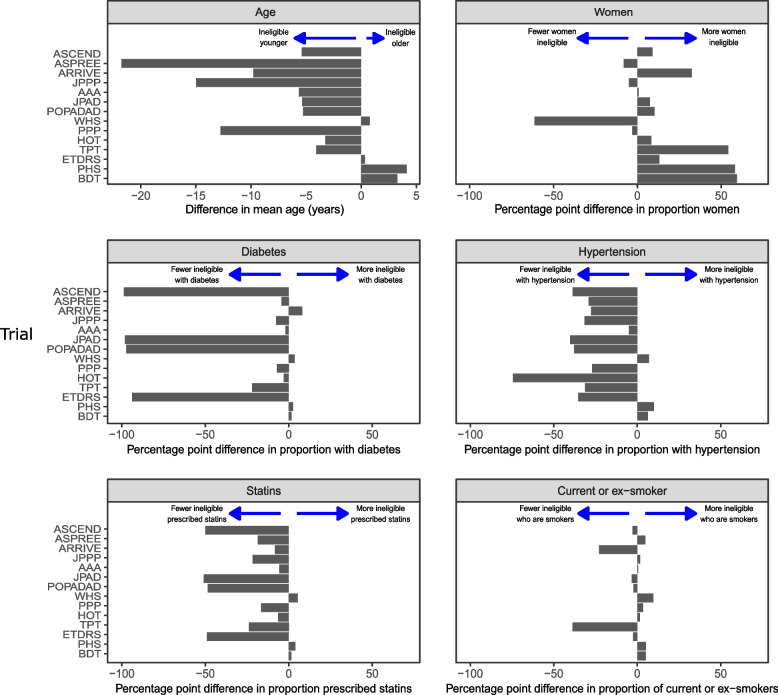
Difference in demography and clinical characteristics in each trial according to eligibility status. Eligibility measured in the whole cohort of adults aged 40 years and over. Trials in chronological order, newest at the top

Figure 3 and Additional file 1: Table S16 detail the differences between the characteristics of trial-ineligible and trial-eligible in the whole cohort of adults aged 40 years and over. In ten trials, the trial-ineligible population was younger than the trial-eligible population and in three trials the trial-ineligible population was > 10 years younger. The difference in mean age between trial-ineligible and trial-eligible populations ranged from 4.1 years older in the Physician’s Health Study (PHS) to 21.7 years younger in ASPirin in Reducing Events in the Elderly (ASPREE). Three trials excluded all women: PHS, British Doctor’s Trial (BDT), and Thrombosis Prevention Trial (TPT). One trial excluded all men: Women’s Health Study (WHS). In seven of the remaining ten trials, a greater percentage of women were ineligible. In 11 trials, the trial-ineligible populations had a lower percentage of people with at least three common cardiovascular risk indicators (diabetes, hypertension, a regular statin prescription, and a smoking history); however, in the three remaining trials (BDT, PHS, and WHS), all four were present in a greater percentage of trial-ineligible populations. Additional file 1: Table S16 also shows comparisons for age and sex between the eligible populations from the whole cohort of everyone aged 40 years or older in CPRD and those recruited to the trials. Out of the 11 trials which reported mean age, eligible populations were generally slightly younger than trial populations (median difference: − 1.2 years, IQR − 3.0–1.1) and out of the 10 trials which recruited people of both sexes, eligible populations generally had more women than trial populations (median percentage point difference: 2.7, IQR − 0.3–6.3).

Table 3 summarises differences between eligible and ineligible populations across all trials, and Additional file 1: Table S17 details differences in each trial.

**Table 3:** Summary characteristics of trial-eligible and trial-ineligible populations in the whole cohort of adults aged 40 years and over for 14 trials

Characteristic in each trial	Ineligible Median (IQR)^a^	Eligible Median (IQR)^a^	***p*** value^b^
Mean age (years)	57.8 (55.5–58.1)	62.6 (58.5–65.6)	0.008
Proportion women	53.1% (51.9–54.0)	44.4% (27.4–54.1)	0.085
Proportion from minority ethnic group	5.2% (5.0–5.4)	4.1% (3.3–8.2)	0.210
Proportion with moderate or severe frailty	7.3% (6.5–7.9)	6.7% (3.9–18.4)	0.765
Proportion care home resident	0.5% (0.4–0.5)	0.1% (0.1–0.2)	< 0.001
Mean BMI (kg/m^**2**^)	27.4 (27.3–27.5)	28.3 (27.3–30.9)	0.033
Proportion with diabetes	6.4% (5.0–7.4)	11.5% (6.4–82.2)	0.015
Proportion with hypertension	23.9% (23.1–25.5)	50.9% (31.9–61.7)	0.004
Proportion current or ex-smoker	38.2% (37.8–38.6)	37.3% (34.3–40.6)	0.635
Proportion prescribed statins	11.8% (10.3–13.2)	26.7% (16.7–53.9)	0.001
Proportion prescribed aspirin	2.5% (1.9–2.7)	5.0% (2.6–11.3)	0.023
Proportion prescribed OACs	1.8% (1.8–2.2)	0.0% (0.0–0.0)	< 0.001
Proportion prescribed NSAIDs	3.0% (2.9–3.1)	3.5% (0.4–4.6)	0.462
Proportion prescribed SSRIs	6.3% (6.2–6.6)	5.2% (3.7–7.3)	0.260

*BMI* body mass index, *IQR* interquartile range, *OAC* oral anticoagulant, *NSAID* non-steroidal anti-inflammatory drug, *SSRI* selective serotonin reuptake inhibitor.

^a^Median and interquartile range calculated at trial level, using mean or proportion among the eligible and ineligible populations from each of the 14 trials

^b^Wilcoxon rank sum test comparing differences between ineligible and eligible populations in each trial

In the whole cohort, trial-ineligible populations were statistically significantly younger (median of all trials’ mean ages 57.8 years for trial-ineligible vs 62.6 years for trial-eligible, *p* = 0.008) but not different in the proportion who were women (median across trials 53.1% vs 44.4%, *p* = 0.085). A greater percentage of trial-ineligible populations lived in a care home (median 0.5 vs 0.1%, *p* < 0.001), but there were no statistically significant differences in terms of ethnicity or frailty. Statistically significantly lower percentages of trial-ineligible populations had hypertension (median 23.9 vs 50.9%, *p* = 0.004), diabetes (median 6.4 vs 11.5%, *p* = 0.015), and were prescribed a statin (median 11.8% vs 26.7%, *p* = 0.001), and trial-ineligible populations had a lower mean BMI (median 27.4 vs 28.3, *p* = 0.033). There were no statistically significant differences in the proportions who were current/ex-smokers. Trial-ineligible populations were less likely to be on aspirin (median 2.5% vs 5.0%, *p* = 0.023) and more likely to be on an oral anticoagulant (median 1.8% vs 0%, *p* < 0.001).

**Fig. 4:**
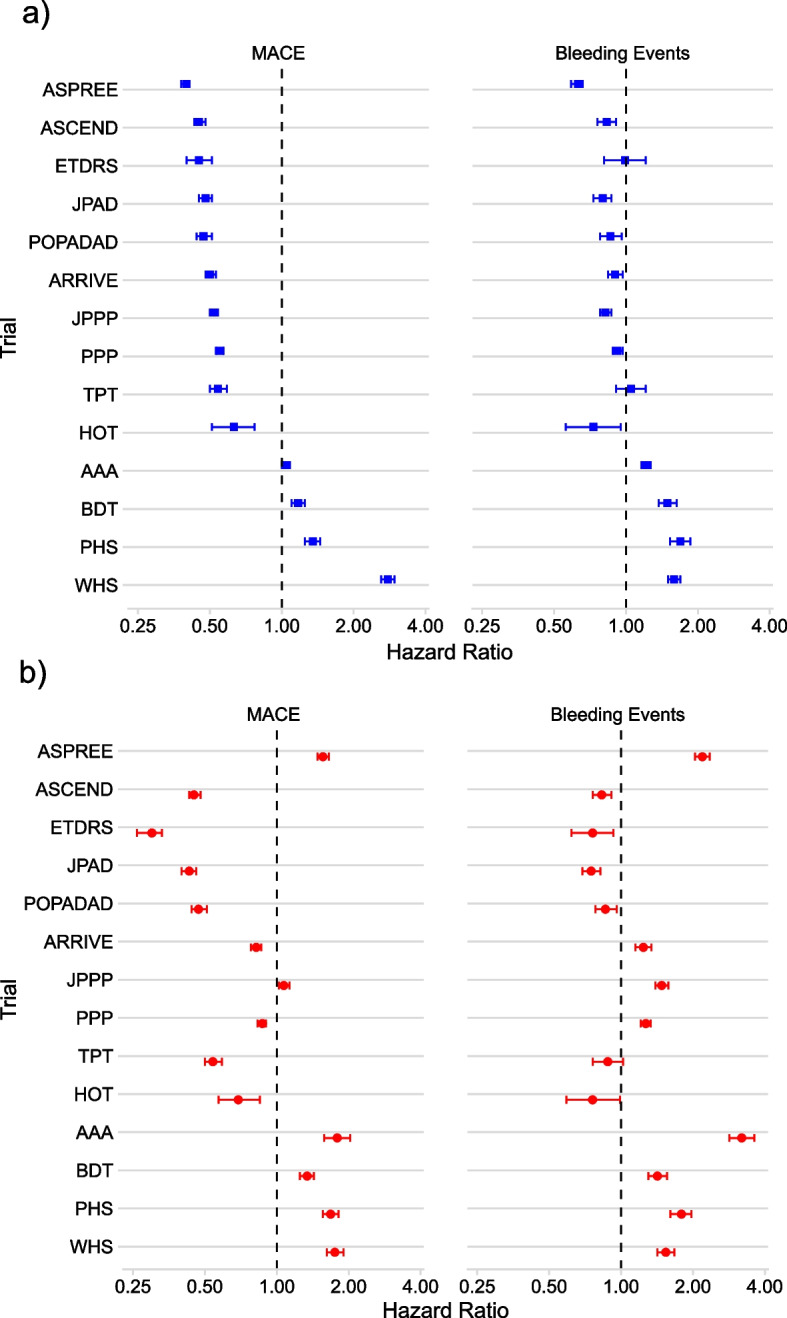
Hazards of MACE and bleeding for trial-ineligible compared with trial-eligible for each trial. MACE, major adverse cardiovascular events. Presented as hazard ratios (HRs) with 95% confidence intervals, for analyses in two different populations: **a** the whole primary prevention population aged over 40 years (blue squares) and **b** the trial-specific age-sex criteria population (red circles). Trials ordered by HR for MACE in the whole cohort. HR < 1 means that trial-ineligible have lower risk than trial-eligible; HR > 1 means that trial-ineligible have higher risk than trial-eligible

**Fig. 5:**
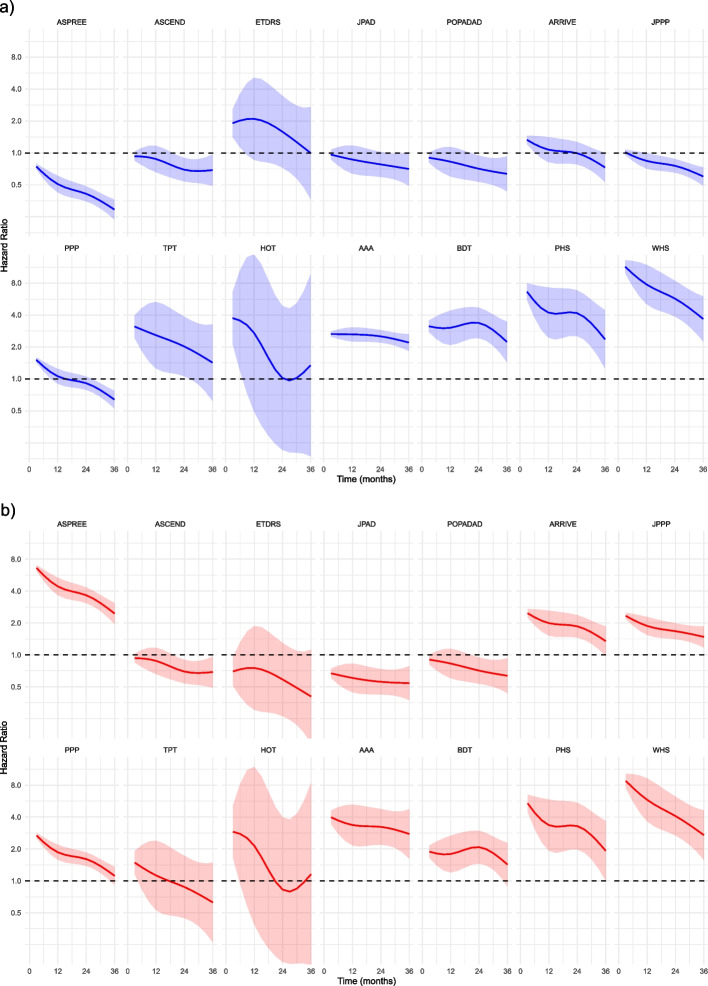
Time-varying hazards of non-cardiovascular death in trial-ineligible compared with trial-eligible for each trial. Presented as quarterly time-varying hazard ratios (HRs) with 95% confidence interval bands measured across 3 years of follow-up, for two different populations: **a** the whole primary prevention population aged 40 years or more (blue) and **b** trial-specific age-sex criteria populations (red). Trials ordered by increasing HR for MACE in the whole cohort. HR < 1 means that trial-ineligible have lower risk than trial-eligible; HR > 1 means that trial-ineligible have higher risk than trial-eligible

For the whole cohort of patients aged 40 years and over without established CVD, 1.9% experienced MACE over 3 years of follow-up, 1.3% experienced major bleeding events, and 3.0% died of non-cardiovascular causes. Median follow-up was 3.0 years. Figures 4 and 5, along with Additional file 1: Tables S18–S19, present the HRs for the whole cohort and trial-specific age-sex criteria populations, comparing trial-ineligible with trial-eligible for MACE and bleeding events (Fig. 4 and Additional file 1: Table S18), as well as the maximum and minimum time-varying HRs for non-CVD death (Fig. 5 and Additional file 1: Table S19).

In the whole cohort, HRs for MACE in the trial-ineligible ranged from 0.40 (95%CI 0.38–0.41) in ASPREE to 2.78 (95%CI 2.61–2.96) in WHS, with trial-ineligible populations having statistically significantly lower hazard of MACE in ten trials and a statistically significant higher HR in four (Fig. 4a). Hazard ratios for major bleeding events in the whole cohort ranged from 0.63 (95%CI 0.59–0.66) in ASPREE to 1.69 (95%CI 1.53–1.86) in PHS, with a statistically significantly lower hazard of major bleeding in the trial-ineligible in eight trials, no difference in two trials, and higher in four (Fig. 4a).

Hazard ratios for non-CVD death were > 1 throughout follow-up for five trials (i.e. higher rate of non-CVD death in trial-ineligible) and < 1 throughout follow-up for four, while in the five remaining trials the general pattern was of higher hazards of non-CVD death early but the same or lower later in follow-up (Fig. 5a).

In the trial-specific age-sex criteria analyses (only analysing patients satisfying each trial’s age-sex inclusion criteria), HRs for MACE in trial-specific age-sex criteria populations ranged from 0.30 (95%CI 0.26–0.51) in ETDRS to 1.79 (95%CI 1.58–2.03) in AAA (Fig. 4b). The hazard of MACE remained statistically significantly lower in the trial-ineligible in eight trials and higher in six. Compared to the whole cohort analyses, the direction of association with MACE reversed for two trials, most notably for ASPREE (HR in whole cohort 1.56 compared to HR in trial-specific age-sex criteria populations 0.40). Hazard ratios for major bleeding events ranged from 0.75 (95%CI 0.69–0.82) in JPAD to 2.19 (95%CI 2.04–2.35) in ASPREE. There was a statistically significantly higher hazard of major bleeding in the trial-ineligible in eight out of the 14 trials, with five trials exhibiting a statistically significantly lower hazard of bleeding.

For non-CVD mortality, HRs were > 1 throughout follow-up (i.e. higher rate of non-CVD death in trial-ineligible) for eight trials, < 1 throughout follow-up for four trials, and no different or variable for two (Fig. 5b). In many trials, the size of HRs > 1 declined with longer follow-up (the ineligible become more similar to the eligible in terms of non-CVD death), consistent with the ineligible having higher initial non-CVD mortality.

## Discussion

One-quarter of all adults aged 40 years and over without cardiovascular disease were ineligible for all 14 trials of aspirin for primary prevention, and more than half were ineligible for at least 12 trials. The trial-ineligible were usually younger, had a lower BMI, had lower rates of diabetes, hypertension, and regular statin prescriptions, but were more likely to be a care home resident. Although there is some variation between trials, these differences reflect the general pattern of inclusion/exclusion criteria to explicitly exclude older people (with an explicit age threshold in eight trials) and a more complex interplay of inclusion criteria selecting people at higher CVD risk and exclusion criteria removing people at higher risk of adverse events. Ineligibility for individual trials was somewhat lower, but still substantial, in more narrowly defined populations (including people at higher predicted cardiovascular risk and people who fitted each trial’s age and sex criteria).

In the whole cohort of adults aged 40 years and over, trial-ineligible populations in ten trials had a lower risk of MACE when compared with the trial-eligible (reflecting the fact that trial selection was often for people at higher CVD risk). When focussing only on patients within the age-sex strata of each trial, the hazards for MACE were higher in the trial-ineligible in two additional trials. Conversely, HRs of bleeding were often higher in ineligible populations after trial-specific age-sex criteria were applied to them, likely reflecting the disproportionate exclusion of individuals at highest risk of adverse events. Similarly, HRs of non-cardiovascular death were generally higher among the ineligible, particularly earlier in follow-up which likely reflects exclusion of people with life-limiting illnesses.

The ASPREE trial provides a particularly interesting example of selection (Figs. 4 and 5). In the whole population analysis, ASPREE trial-eligible had higher hazards of MACE, bleeding, and non-CVD death. However, when focussing only on patients within the age-sex strata of each trial, ASPREE trial-eligible had notably lower hazards of MACE, bleeding, and non-CVD death. ASPREE therefore appeared to select higher risk patients through its age criteria, but within the older population selected, systematically selected lower risk patients. This suggests that conducting a trial in an older population does not mean findings are applicable to all older people.

Previous studies have found that older people and women are disproportionately ineligible for trials of many treatments [58–61]. In contrast, in our study, the trial-ineligible were generally younger, likely reflecting selection of people at higher CVD risk. Across all trials, there was no statistically significant difference in the median proportion of women who were ineligible, despite three early trials excluding all women. This was counteracted by more equitable eligibility of women in later trials, including WHS which excluded all men.

A study examining eligibility for trials of antiplatelets for secondary prevention also found that women were more likely to be ineligible but that trial-ineligible were consistently older [62]. Previous studies analysing eligibility for individual trials of statins for primary prevention found that ineligibility was also high, ranging from 69.2 to 99.6% [63, 64]. Like early trials of aspirin, many early trials of statins excluded all women, but in contrast to this study, disproportionate ineligibility of women for statin trials continued, with 48.2% of women ineligible for all 11 trials in the most recent study, compared with 28.3% of men [64]. Both studies also found that statin trial-ineligible populations had lower rates of CVD events, with a third study showing a strong positive correlation between 10-year CVD risk and eligibility for statin trials [65], aligning with our findings that those with higher risk are better represented. None of these studies analysed adverse events or competing mortality, nor directly compared characteristics of the trial-ineligible and trial-eligible.

Studies of trials in other CVD conditions have shown the trial-ineligible to be younger and to have comparable or lower rates of trial target outcomes. In a study of oral anticoagulant trials in people with atrial fibrillation, rates of stroke or systemic embolism were similar in the whole comparison population compared with the trial-eligible [66]. In contrast, in a recent study of eligibility for contemporary heart failure trials, trial-ineligible populations experienced lower rates of heart failure hospitalisation and cardiovascular death, reflecting the selection of people with the most severe disease [67]. While this study did not adjust for confounders, they noted that there were proportionately higher rates of non-cardiovascular deaths compared with cardiovascular deaths in the trial-ineligible. This echoes our findings, which we interpret as trials often selecting people at higher CVD risk, but with lower bleeding risk or competing mortality risk.

Strengths of the study include examination in a large representative population of the impact of trial eligibility criteria for all the trials that underpin guideline recommendations on using aspirin for primary prevention of CVD. Previous eligibility studies have generally focussed on a single trial or a handful of trials, whereas we systematically selected all trials which contribute evidence to multiple international recommendations. The study population has low use of aspirin for primary prevention, with 2.6% of the cohort regularly prescribed it, reflecting current guidelines in England [14] and a recent Danish study [68]. This means that most differences between trial-eligible and trial-ineligible populations are unlikely to be meaningfully confounded by aspirin treatment.

The study also has several limitations. The key limitation is that we can only model explicit inclusion and exclusion criteria, and it is very likely that implicit criteria (often framed in terms of ‘investigator judgement’ or ‘discretion’) impose additional selection. Ineligibility will therefore be underestimated. Comparison of ineligibility between trials should also be cautious, because lower ineligibility may reflect either less precisely defined (more implicit) eligibility criteria, explicit criteria which cannot be mapped to routine data, or both. A notable example was the AAA trial, which had the lowest proportion estimated as trial-ineligible (42.5% of everyone aged 40 years or older). This trial had few explicit inclusion/exclusion criteria, but a key explicit criterion (an ABPI of ≤ 0.95 in at least one limb), which would exclude a large proportion of patients, could not be mapped because it is very rarely recorded in routine data. Exclusion will therefore likely be underestimated, with potential bias towards finding no difference between eligible and ineligible. Conversely, because we undertook our analysis with a broad definition of the primary prevention population, including those with lower cardiovascular risk in our analyses may underestimate adverse outcomes in the ineligible and overestimate ineligibility, particularly in trials designed to investigate very specific subsets of patients, such as ETDRS examining people with diabetic retinopathy. However, our primary aim was to examine how the evidence from each trial applied to the whole cohort of adults aged 40 years and over without established cardiovascular disease, since this is the implied target population of published meta-analyses of the effectiveness of aspirin for primary prevention which include all trials. The comparisons in the whole cohort represent the differences between ineligible and eligible from this perspective, where age and sex exclusions are often important contributors to ineligibility. We additionally report comparisons focussing on the subpopulations defined by each trial’s age and sex criteria, which explores differences arising from *other* eligibility criteria. Outcomes were only measured for a period of 3 years, which is shorter than all 14 trials (Additional file 1: Table S1). However, using this timeframe, we were able to demonstrate statistically significant and meaningful differences. Finally, the improvement in CVD risk reduction strategies such as increased prescribing of statins and antihypertensives may have changed the CVD risk profile in the present compared to when our data were captured at the end of 2015.

Aspirin is no longer recommended for primary prevention in many countries, but recent USPSTF guidance recommends considering prescribing aspirin for primary prevention in people between the ages of 40 and 59 years at high CVD risk. Furthermore, although aspirin use for primary CVD prevention is declining in the USA, more than one in six Americans aged 40 years or older without CVD take such aspirin [69], three-quarters of which is on a doctor’s recommendation [70]. Most RCTs we evaluated concluded that the benefits of aspirin, in terms of reducing cardiovascular events, were closely balanced against the risk of harm from bleeding events. Our results show that, for most trials, people at lower risk of CVD (younger, with fewer CVD risk factors) and those at higher risk of bleeding and non-CVD mortality were selected out with 24% ineligible for all trials. However, even with this patient selection, the trials provided no convincing evidence of an overall net benefit. It is therefore unlikely that any previously excluded subgroup would derive benefit. Furthermore, risk stratification using scores is unlikely to improve much for the whole population, even with the addition of novel blood tests [71, 72] or genetic testing [73]. However, screening by imaging to identify asymptomatic atherosclerosis is a plausibly effective alternative strategy to determine treatment with aspirin and other preventive drugs like statins, where the findings of ongoing research will be valuable [74].

While this study focusses solely on aspirin for primary prevention of CVD, more research is needed for preventative treatments for both primary and secondary prevention. While it would be expected that the evidence in patients following MI would be strong and generalisable, there may be more value in exploring applicability of evidence for aspirin in conditions such as stable angina, where trial evidence is more limited and older, and likely has greater applicability concerns.

## Conclusions

Most people would have been ineligible for individual trials of aspirin for the primary prevention of CVD, although three-quarters would have been eligible for at least one trial. Trials selected patients with a wide range of cardiovascular risk, with trials in people at higher cardiovascular risk often also selecting people at higher risk of bleeding and, in some cases, non-cardiovascular death than younger, trial-ineligible populations. However, when comparing people in the same age and sex strata as the trial-eligible, people at higher risk of bleeding and non-cardiovascular death were more likely to be ineligible for most trials. This selection of participants at lower risk of harm or competing mortality suggests that many trials may overestimate net benefit in the clinical population without established CVD. More explicit consideration of the applicability of trial evidence in guideline development would be beneficial, and identifying populations systematically excluded from trials will highlight patient groups to whom guideline recommendations may not apply.

## Supplementary Information

Additional file 1. Tables S1–S19. Table S1 Trial characteristics. Tables S2–S15 Eligibility criteria for each of the 14 trials. Table S16 Differences between trial-eligible, trial-ineligible, and trial participants. Table S17 Differences between trial-eligible and trial-ineligible for each of the 14 trials. Table S18 Hazards of MACE and bleeding events. Table S19 Time-varying hazards of non-CVD death.

## Data Availability

The authors cannot directly share the data because they were used under license for the current study, and the licence does not permit sharing. The data are available to any researcher by application to Clinical Practice Research Datalink ([https://www.cprd.com/](https:/www.cprd.com)).

## Abbreviations

AAA: Aspirin for Asymptomatic Atherosclerosis
ASPREE: ASPirin in Reducing Events in the Elderly
BDT: British Doctor’s Trial
BMI: Body mass index
CCI: Charlson Comorbidity Index
CPRD: Clinical Practice Research Datalink
CVD: Cardiovascular disease
eFI: Electronic Frailty Index
ETDRS: Early Treatment Diabetic Retinopathy Study
JPAD: Japanese Primary prevention of Atherosclerosis with aspirin for Diabetes
GP: General practitioner
HES: Hospital Episode Statistics
HOT: Hypertension Optimisation Trial
HR: Hazard ratio
MACE: Major adverse cardiovascular event
MI: Myocardial infarction
NHS: National Health Service
ONS: Office for National Statistics
PAD: Peripheral arterial disease
PHS: Physician’s Health Study
POPADAD: Prevention Of Progression of Arterial Disease And Diabetes
RCT: Randomised clinical trial
TIA: Transient ischaemic attack
TPT: Thrombosis Prevention Trial
UK: United Kingdom
USPSTF: United States Preventative Services Task Force
WHS: Women’s Health Study
