# Supplementary material for: Eligibility of real-world patients for aspirin primary prevention trials in cardiovascular disease
Source: BMC Med. 2026 Jan 27;24:57. doi: 10.1186/s12916-026-04654-w (PMC12857110; doi:10.1186/s12916-026-04654-w)
Supplement: Supplementary file 1 — Additional file 1. Tables S1–S19. Table S1 Trial characteristics. Tables S2–S15 Eligibility criteria for each of the 14 trials. Table S16 Differences between trial-eligible, trial-ineligible, and trial participants. Table S17 Differences between trial-eligible and trial-ineligible for each of the 14 trials. Table S18 Hazards of MACE and bleeding events. Table S19 Time-varying hazards of non-CVD death. [file 12916_2026_4654_MOESM1_ESM.docx]

## Additional File 1

Holder M, Morales DR, Hanlon P, McAllister D, Guthrie B. Eligibility of real-world patients for aspirin primary prevention trials in cardiovascular disease

**Contents**

[Table S1. Trial characteristics 2](#_Toc218605278)

[Table S2. Eligibility criteria for British Doctors’ Trial (BDT) 4](#_Toc218605279)

[Table S3. Eligibility criteria for Physicians’ Health Study (PHS) 5](#_Toc218605280)

[Table S4. Eligibility criteria for Early Treatment Diabetic Retinopathy Study (ETDRS) 7](#_Toc218605281)

[Table S5. Eligibility criteria for Hypertension Optimal Treatment (HOT) 9](#_Toc218605282)

[Table S6. Eligibility criteria for Thrombosis Prevention Trial (TPT) 11](#_Toc218605283)

[Table S7. Eligibility criteria for Primary Prevention Project (PPP) 13](#_Toc218605284)

[Table S8. Eligibility criteria for Women’s Health Study (WHS) 16](#_Toc218605285)

[Table S9. Eligibility criteria for Prevention Of Progression of Arterial Disease And Diabetes (POPADAD) 18](#_Toc218605286)

[Table S10. Eligibility criteria for Japanese Primary prevention of Atherosclerosis with aspirin for Diabetes (JPAD) 20](#_Toc218605287)

[Table S11. Eligibility criteria for Aspirin for Asymptomatic Atherosclerosis (AAA) 22](#_Toc218605288)

[Table S12. Eligibility criteria for Japanese Primary Prevention Project (JPPP) 23](#_Toc218605289)

[Table S13. Eligibility criteria for Aspirin to Reduce the Risks of Initial Vascular Events (ARRIVE) 26](#_Toc218605290)

[Table S14. Eligibility criteria for A Study of Cardiovascular Events iN Diabetes (ASCEND). 31](#_Toc218605291)

[Table S15. Eligibility criteria for ASPirin for Reducing Events in the Elderly (ASPREE). 34](#_Toc218605292)

[Table S16. Comparisons between trial-eligible in CPRD Gold population, trial-ineligible in CPRD Gold population, and people recruited to trial. 37](#_Toc218605293)

[Table S17. Differences in characteristics between the trial-eligible and trial-ineligible in each trial. 38](#_Toc218605294)

[Table S18. Hazards of major adverse cardiovascular events and bleeding events in the trial ineligible compared with the trial-eligible 43](#_Toc218605295)

[Table S19. Time varying hazards of non-cardiovascular deaths in the trial ineligible compared with the trial-eligible 44](#_Toc218605296)

1

# Table S1. Trial characteristics

| First Author | Trial | Location(s) | Average follow up (years: method) | Target population (n) | Intervention | Control |
| --- | --- | --- | --- | --- | --- | --- |
| Peto (1988) [27] | British Doctor’s Trial (BDT) | UK | 5.1: mean | “healthy male doctors” (5139) | Aspirin 500mg/300mg OD | No treatment |
| Steering Committee of the PHS (1989) [28] | Physician’s Health Study (PHS) | USA | 5.0 | “healthy male physicians” (22071) | Aspirin 325mg alternate days ± beta carotene | Placebo |
| ETDRS Study Group (1992) [29] | Early Treatment Diabetic Retinopathy Study (ETDRS) | USA | 5.0 | “a clinical diagnosis of diabetes mellitus and … diabetic retinopathy” (3711) | Aspirin 650mg OD | Placebo |
| Meade (1998) [30] | Thrombosis Prevention Trial (TPT) | UK | 6.8: median | “men aged between 45 and 69 years at high risk of IHD” (5085 at aspirin stage) | Aspirin 75mg OD ± warfarin (INR 1.5) | Placebo |
| Hansson (1998) [31] | Hypertension Optimal Treatment (HOT) | 26 countries | 3.8 | “aged 50-80 years with hypertension and diastolic blood pressure between 100 mm Hg and 115 mm Hg” (18790) | Blood pressure targets and aspirin 75mg OD | Placebo |
| De Gaetano (2001) [32] | Primary Prevention Project (PPP) | Italy | 3.6: mean, 4.0: median | “aged 50 years or greater, with at least one of the major recognised cardiovascular risk factors.” (4495) | Aspirin 100mg OD ± vitamin E | Placebo |
| Ridker (2005) [33] | Women’s Health Study (WHS) | USA | 10.1 | “healthy [female healthcare professionals] 45 years of age or older” (39876) | Aspirin 100mg alternate days ± vitamin E ± beta-carotene | Placebo |
| Belch (2008) [34] | Prevention Of Progression of Arterial Disease And Diabetes (POPADAD) | UK | 6.7: median | “patients with diabetes mellitus and asymptomatic peripheral arterial disease” (1276) | Aspirin 100mg OD ± antioxidant | Placebo |
| Ogawa (2008) [35] | Japanese Primary prevention of Atherosclerosis with aspirin for Diabetes (JPAD) | Japan | 4.4: median | “patients with type 2 diabetes without a history of atherosclerotic disease” (2539) | Aspirin 100mg/81mg OD | No treatment |
| Fowkes (2010) [36] | Aspirin for Asymptomatic Atherosclerosis (AAA) | UK | 8.2: mean | “aged 50 to 75 living in central Scotland, free of clinical cardiovascular disease … with a low ABI” (3350) | Aspirin 100mg OD | Placebo |
| Ikeda (2014) [6] | Japanese Primary Prevention Project (JPPP) | Japan | 5.0: median | “aged 60 to 85 years, presenting with hypertension, dyslipidaemia, or diabetes mellitus” (14464) | Aspirin 100mg OD | No treatment |
| Gaziano (2018) [13] | Use of Aspirin to Reduce Risk of Initial Vascular Events in patients at moderate risk of cardiovascular disease (ARRIVE) | 7 countries | 5.0: median | “55 years (men) or 60 years (women) and older and had an average cardiovascular risk … [and without] a high risk of … bleeding, or diabetes.” (12546) | Aspirin 100mg OD | Placebo |
| McNeil (2018) [7] | ASPirin in Reducing Events in the Elderly (ASPREE) | Australia, USA | 4.7: median | “70 years of age or older (or ≥65 years of age among blacks and Hispanics in the United States) and did not have cardiovascular disease, dementia, or disability.” (19114) | Aspirin 100mg OD | Placebo |
| Bowman (2018) [12] | A Study of Cardiovascular Events iN Diabetes (ASCEND) | UK | 7.4: mean | “at least 40 years of age … [with] a diagnosis of diabetes mellitus (any type) and did not have known cardiovascular disease” | Aspirin 100mg OD | Placebo |

# Table S2. Eligibility criteria for British Doctors’ Trial (BDT)

| Trial Register | Protocol, Baseline or Preliminary Results Paper | Paper [27] | Criterion for matching | Rule for dataset^*^ |
| --- | --- | --- | --- | --- |
| **Inclusion** | **Inclusion** | **Inclusion** | **Inclusion** | **Inclusion** |
| N/A | N/A | “In 1978…all male doctors resident in the United Kingdom who were born this century” | Male doctors aged <79 | Men  Aged <79  IMD top quintile [38] |
| **Exclusion** | **Exclusion** | **Exclusion** | **Exclusion** | **Exclusion** |
| N/A | N/A | “…already taking aspirin for various reasons” | Prescribed aspirin | Not implemented |
|  |  | “…could not take [aspirin]” | Aspirin allergy or contraindicated | Any aspirin contraindication:  Primary thrombocytopaenia ever  Coagulation defects ever  Hospital admission with peptic ulcer (first position) in last 12 months |
|  |  | “…a history of peptic ulcer, stroke or definite myocardial infarction” | History of:  Peptic ulcer  Stroke  MI | Peptic ulcer ever  Any ICH ever  Not implemented |

*ICH = intracranial haemorrhage, IMD = index of multiple deprivation, MI = myocardial infarction*

**In all studies, patients with a history of acute coronary syndrome, myocardial infarction, angina, peripheral arterial disease, ischaemic stroke or transient ischaemic attack were excluded at the point of data extraction.*

# Table S3. Eligibility criteria for Physicians’ Health Study (PHS)

| Trial Register [39] | Preliminary Findings Paper [40] | Paper [28] | Criterion for matching | Rule for dataset^*^ |
| --- | --- | --- | --- | --- |
| **Inclusion** | **Inclusion** | **Inclusion** | **Inclusion** | **Inclusion** |
| “Male physicians ages 40 to 84” | “Male physicians aged 40-84 residing in the United States” | Not stated | Male physicians aged 40-84 | Men  Aged 40-84  IMD top quintile [41] |
| “No history of stroke, MI, cancer or renal disease” |  |  | N/A – See Exclusion |  |
| “No contraindications to aspirin or beta-carotene” |  |  | N/A – See Exclusion |  |
| “No current usage of aspirin or vitamin A tables greater than once per week” |  |  | N/A – See Exclusion |  |
| **Exclusion** | **Exclusion** | **Exclusion** | **Exclusion** | **Exclusion** |
| None | “personal history of MI, stroke, TIA, cancer (except non-melanoma skin cancer) current liver or renal disease, peptic ulcer, or gout” | Not stated | History of:  MI  Stroke  TIA  Cancer (excluding non-melanoma skin cancer)  Active liver disease  Active renal disease  Peptic ulcer  Gout | Not implemented  Any ICH ever  TIA ever  Cancer (excl non-melanoma skin) ever  Any of the following ever: alcoholic liver disease, autoimmune liver disease, chronic viral hepatitis, cirrhosis, liver failure, portal hypertension, oesophageal varices. Any of the following at last measurement: ALT>3xULN, AST>3xULN, bilirubin>3xULN  History of CKD 3 or worse (last eGFR recorded), no end-stage renal disease ever.  Peptic ulcer ever  Gout ever |
|  | “contraindications to aspirin consumption” |  | Aspirin contraindicated | Any aspirin contraindication:  Primary thrombocytopaenia ever  Coagulation defects ever  Hospital admission with peptic ulcer (first position) in last 12 months |
|  | “current use of aspirin, other platelet-active drugs, or nonsteroidal anti-inflammatory agents” |  | Prescribed:  Aspirin  Antiplatelet drugs  NSAIDs | Not implemented  Not implemented  Current NSAID |
|  | “current use of a vitamin A supplement” |  | Prescribed Vitamin A supplement | Not implemented |
|  | “inadequate compliance (defined as taking less than two thirds of their pills [during run-in phase])” |  | Inadequate compliance | Not implemented |

*ALT = alanine transaminase, AST = aspartate transaminase, CKD = chronic kidney disease, eGFR = estimated glomerular filtration rate, ICH = intracranial haemorrhage, IMD = index of multiple deprivation, MI = myocardial infarction, NSAID = non-steroidal anti-inflammatory drug, TIA = transient ischaemic attack, ULN = upper limit of normal*

**In all studies, patients with a history of acute coronary syndrome, myocardial infarction, angina, peripheral arterial disease, ischaemic stroke or transient ischaemic attack were excluded at the point of data extraction.*

# Table S4. Eligibility criteria for Early Treatment Diabetic Retinopathy Study (ETDRS)

| Trial Register [42] | Baseline [43] | Paper [29] | Criterion for matching | Rule for dataset^*^ |
| --- | --- | --- | --- | --- |
| **Inclusion** | **Inclusion** | **Inclusion** | **Inclusion** | **Inclusion** |
| “Men and women between the ages of 18 and 70 years with moderate or severe nonproliferative diabetic retinopathy or mild proliferative retinopathy in both eyes with no previous photocoagulation treatment and with visual acuity of 20/40 or better (20/200 or better if macular edema is present)” | N/A – manual referenced but unable to source | “A clinical diagnosis of diabetes mellitus and one of the following categories of diabetic retinopathy: mild non-proliferative with macular edema, or moderate to severe non-proliferative or early proliferative (less severe than the high-risk proliferative stage, as defined by the Diabetic Retinopathy Study) with or without macular edema” | Aged 18-70  Diagnosis of diabetes mellitus  Diagnosis of diabetic retinopathy  No previous photocoagulation | Aged 18-70  Diabetes mellitus (any type) ever  Diabetic retinopathy ever  Not implemented |
|  |  | “Visual acuity was required to be better than 20/40 in each eye (or 20/400 if acuity was reduced as a result of diabetic macular edema)” | Visual acuity better than 20/40 | Absence of blindness |
| **Exclusion** | **Exclusion** | **Exclusion** | **Exclusion** | **Exclusion** |
| None | N/A | “Systolic blood pressure over 210 mmHg and/or diastolic over 110 mmHg despite the use of antihypertensive medication” | Prescribed antihypertensives  AND  (SBP > 210 mmHg  OR  DBP >110 mmHg) | Current thiazide or current beta-blocker or current ACE-inhibitor or current ARB or current calcium channel blocker or current cardiac alpha channel blocker  AND  (SBP>210  OR  DBP>110) |
|  |  | “history of gastrointestinal hemorrhage or diagnosis of active gastrointestinal ulcer in past 2 years” | History of (in the past 2 years):  GI bleed  Active GI ulcer | Hospital admission with GI bleed (primary position) last 2 years  Hospital admission with GI ulcer last 2 years  Hospital admission with GI ulcer complications (e.g. perforation) in last 2 years |
|  |  | “inability or unwillingness to stop taking anticoagulants or anti-platelet drugs” | Prescribed:  Anticoagulants  Antiplatelets | Current OAC  Not implemented |
|  |  | “allergy to aspirin” | Aspirin allergy | Not implemented |
|  |  | “pregnancy or lactation” | Pregnancy | Not implemented |
|  |  | “poor prognosis for 5 years of follow-up because of a prior major cardiovascular event, cancer or another chronic disease” | Life limiting illness (<5 year prognosis) | Severe frailty  Cancer with poor prognosis – ovary, stomach, lung, oesophagus, liver, brain, pancreas, mesothelioma or any secondary  Major chronic illness – pulmonary hypertension, cirrhosis, portal hypertension, dementia.  Hospital admission with heart failure, COPD or PD  CCI >5 |

*ACE = angiotensin-converting enzyme, ARB = angiotensin II receptor blocker, CCI = Charlson comorbidity index, COPD = chronic obstructive pulmonary disease, DBP = diastolic blood pressure, GI = gastrointestinal, OAC = oral anticoagulant, PD = Parkinson’s disease, SBP = systolic blood pressure*

**In all studies, patients with a history of acute coronary syndrome, myocardial infarction, angina, peripheral arterial disease, ischaemic stroke or transient ischaemic attack were excluded at the point of data extraction.*

# Table S5. Eligibility criteria for Hypertension Optimal Treatment (HOT)

| Trial Register | Protocol Paper [44] | Paper [31] | Criterion for matching | Rule for dataset^*^ |
| --- | --- | --- | --- | --- |
| **Inclusion** | **Inclusion** | **Inclusion** | **Inclusion** | **Inclusion** |
| N/A | “Hypertensive men and women between 50 and 80 years of age with essential hypertension… Their diastolic blood pressure must be ≥100 mmHg and ≤115 mmHg on two occasions, at least 1 week apart” | Not stated | Aged 50-80  Essential hypertension  DBP between 100-115 mmHg (inclusive) on 2 occasions, ≥1 week apart | Aged 50-80  Hypertension ever  Last DBP 100-115mmHg |
| **Exclusion** | **Exclusion** | **Exclusion** | **Exclusion** | **Exclusion** |
| N/A | “Malignant hypertension” | Not stated | Malignant hypertension | Not implemented |
|  | “Secondary hypertension” |  | Secondary hypertension | eGFR <30  ESRD ever |
|  | “Diastolic BP >115 mmHg” |  | DBP >115 mmHg | (Duplicate) |
|  | “Stroke or myocardial infarction within 12 months prior to randomization” |  | History of (in the past 12 months):  Stroke  MI | Any ICH in last 12 months  Not implemented |
|  | “Decompensated congestive heart failure” |  | Decompensated heart failure | Hospital admission with heart failure ever |
|  | “Other serious concomitant disease which, in the opinion of the investigator, could affect survival during the next 2-3 years” |  | Life limiting illness (<2 year prognosis) | Severe frailty  Cancer with poor prognosis – ovary, stomach, lung, oesophagus, liver, brain, pancreas, mesothelioma or any secondary  Major chronic illness – pulmonary hypertension, cirrhosis, portal hypertension, dementia.  Hospital admission with heart failure, COPD or PD  CCI >5 |
|  | “Patients who, in the opinion of the investigator, require a β-blocker, ACE-inhibitor or diuretic for reasons other than hypertension” |  | Prescribed, for a reason other than hypertension:  β-blocker  ACE-inhibitor  Diuretic | Heart failure ever |
|  | “Patients who, in the opinion of the investigator, require antiplatelet or anticoagulant treatment” |  | Prescribed:  Antiplatelets  Anticoagulants | Not implemented  Current OAC |
|  | “Insulin-treated diabetics” |  | Diabetes mellitus  AND  Taking insulin | Current insulin |
|  | “Patients with known hypersensitivity to felodipine” |  | Allergy to felodipine | Not implemented |
|  | “Patients with known contraindications to low-dose ASA” |  | Aspirin contraindicated | Any aspirin contraindication:  Primary thrombocytopaenia ever  Coagulation defects ever  Hospital admission with peptic ulcer (first position) in last 12 months |

*ACE = angiotensin-converting enzyme, ASA = acetylsalicylic acid, CCI = Charlson comorbidity index, COPD = chronic obstructive pulmonary disease, DBP = diastolic blood pressure, eGFR = estimated glomerular filtration rate, ESRD = end stage renal disease, ICH = intracranial haemorrhage, MI = myocardial infarction, OAC = oral anticoagulant, PD = Parkinson’s disease*

**In all studies, patients with a history of acute coronary syndrome, myocardial infarction, angina, peripheral arterial disease, ischaemic stroke or transient ischaemic attack were excluded at the point of data extraction.*

# Table S6. Eligibility criteria for Thrombosis Prevention Trial (TPT)

| Trial Register [45] | Pilot Study Paper [46] | Paper [30] | Criterion for matching | Rule for dataset^*^ |
| --- | --- | --- | --- | --- |
| **Inclusion** | **Inclusion** | **Inclusion** | **Inclusion** | **Inclusion** |
| “In top 20% of risk score distribution based on smoking history, family history, systolic blood pressure, body mass index, blood cholesterol, factor VII activity, plasma fibrinogen” |  | “those men in the top 20% of the risk score distribution, or in the top 25% in regions with particularly high IHD mortality rates” | “those men in the top 20% of the risk score distribution, or in the top 25% in regions with particularly high IHD mortality rates”  “…based on smoking history, family history, systolic blood pressure, body mass index, blood cholesterol, factor VII activity, plasma fibrinogen” | Men  Risk score calculated using Framingham CVD 10 year risk, including age, BMI, SBP (with or without treatment), smoking and diabetes [47]  Top 20% highest scores out of otherwise eligible included (i.e., this criterion applied last), with top 25% of IMD bottom quintile. |
| “men aged 45-69” | “all men aged between 45 and 64” | “men aged between 45 and 69 years” | Aged 45-69 | Aged 45-69 |
| **Exclusion** | **Exclusion** | **Exclusion** | **Exclusion** | **Exclusion** |
| “Already on antithrombotic treatment” | “already on anti-thrombotic drugs” |  | On antithrombotic treatment | Current OAC |
| “High risk of bleeding” | “a past history of bleeding tendency” |  | High risk or past history of bleeding tendency | Bleeding disorder  Hospital admission with bleeding event ever |
| “Liver or renal disease” | “a range of other conditions including liver disease…” |  | Liver disease  Renal disease | Any of the following ever: alcoholic liver disease, autoimmune liver disease, chronic viral hepatitis, cirrhosis, liver failure, portal hypertension, oesophageal varices. Any of the following at last measurement: ALT>3xULN, AST>3xULN, bilirubin>3xULN  History of CKD 3 or worse (last eGFR recorded), no end-stage renal disease ever. |
| “Serious concomitant disease” | “…and malignant disease, and other illnesses, at the discretion of the general practitioner” |  | Serious or malignant disease | Severe frailty  Any cancer  Major chronic illness – Pulmonary hypertension, cirrhosis, portal hypertension, dementia.  Hospital admission with heart failure, COPD or PD  CCI >5 |
| “At discretion of general practitioner” |  |  | Discretion of GP | Not implemented |
|  | “a past or present history of peptic ulceration, hiatus hernia or oesophagitis, these criteria being rigorously applied and covering a number of men in whom ‘severe indigestion’ was the only symptom recorded” | “current or recent history of possible peptic ulceration” | History of:  Possible peptic ulcer  Hiatus hernia  Oesophagitis  Severe indigestion | Peptic ulcer  Hiatus hernia  Oesophagitis  Indigestion/dyspepsia |
|  | “actual, or likely, regular requirement for drugs interacting with warfarin (with some exceptions)” | “other medication incompatible with trial treatment” | Prescribed (or indication for) drugs which interact with warfarin | Current NSAID  Current SSRI |
|  | “a past history of cerebrovascular disease” | “a history of possible or definite…stroke” | History of cerebrovascular disease | Any ICH ever |
|  | “likely inability to comply with trial requirements” |  | Inability to comply | Not implemented |
|  | “known or suspected alcohol abuse” |  | Known or suspected alcohol abuse | Alcohol misuse ever |
|  | “A past history of IHD (myocardial infarction and/or angina)” | “a history of possible or definite MI…” | History of (possible or definite):  MI  Angina | Not implemented  Not implemented |

*ALT = alanine transaminase, AST = aspartate transaminase, BMI = body mass index, CCI = Charlson comorbidity index, CKD = chronic kidney disease, COPD = chronic obstructive pulmonary disease, CVD = cardiovascular disease, ICH = intracranial haemorrhage, IHD = ischaemic heart disease, IMD = index of multiple deprivation, MI = myocardial infarction, NSAID = non-steroidal anti-inflammatory drug, OAC = oral anticoagulant, PD = Parkinson’s disease, SBP = systolic blood pressure, SSRI = selective serotonin reuptake inhibitor, ULN = upper limit of normal*

**In all studies, patients with a history of acute coronary syndrome, myocardial infarction, angina, peripheral arterial disease, ischaemic stroke or transient ischaemic attack were excluded at the point of data extraction.*

# Table S7. Eligibility criteria for Primary Prevention Project (PPP)

| Trial Register | Trial Protocol Paper [48] | Paper [32] | Criterion for matching | Rule for dataset^*^ |
| --- | --- | --- | --- | --- |
| Inclusion | Inclusion | Inclusion | Inclusion | Inclusion |
| N/A | “patients (of both sexes, 50-75 years old)… [with] the presence of a cardiovascular risk factor… defined as follows” | “both men and women aged 50 years or greater, with at least one of the major recognised cardiovascular risk factors” | Aged ≥50  Satisfies at least one of the following inclusion criteria (in italics) | Aged ≥50  AND  Satisfies ≥1 of following 6 criteria: |
|  | “old age: age ≥65 years” | “old age (≥65 years)” | *Aged ≥65* | Aged ≥65 |
|  | “family history of premature myocardial infarction before 55 years of age in at least one parent or sibling” | “family history of myocardial infarction before 55 years of age in at least one parent or sibling” | *Family history of MI before 55 years of age in parent or sibling* | FH: MI <55 in 1^st^ degree male relative |
|  | “obesity: body mass index ≥30kg/m^2^” | “obesity (body mass index ≥30 kg/m^2^)” | *BMI ≥30 kg/m^2^* | BMI ≥30 kg/m^2^ |
|  | “hypertension: systolic blood pressure ≥160 mmHg or diastolic blood pressure ≥95 mmHg on at least 3 separate occasions, or presence of treatment” | “hypertension (systolic blood pressure [SBP] ≥160 mm Hg or diastolic blood pressure [DBP] ≥95 mm Hg on at least 3 separate occasions) [or chronic drug treatment]” | *Hypertension with drug treatment*  *OR*  *SBP ≥160 mmHg on 3 separate occasions*  *OR*  *DBP ≥95 mmHg on 3 separate occasions* | Hypertension ever AND  (Current thiazide or current beta-blocker or current ACE-inhibitor or current ARB or current calcium channel blocker or current cardiac alpha channel blocker)  OR  SBP ≥160 mmHg  OR  DBP ≥95 mmHg |
|  | “diabetes mellitus: fasting venous plasma glucose concentration ≥7.8 mmol/l on at least two separate occasions, or presence of treatment” | “diabetes mellitus (fasting venous plasma glucose concentration ≥7.8 mmol/L on at least two separate occasions) [or chronic drug treatment]” | *Diabetes mellitus* | Diabetes mellitus |
|  | “hyper-cholesterolaemia: total blood cholesterol levels ≥6.4 mmol/l on at least two separate occasions, or presence of treatment” | “hyper-cholesterolaemia (total blood cholesterol ≥6.4 mmol/L on at least two separate occasions) [or chronic drug treatment]” | *Hyper-cholesterolaemia with drug treatment*  *OR*  *Total blood cholesterol ≥6.4 mmol/L on at least two separate occasions* | Current lipid lowering drugs  OR  Total cholesterol ≥6.4 mmol/l ever |
| **Exclusion** | **Exclusion** | **Exclusion** | **Exclusion** | **Exclusion** |
| N/A | “concomitant diseases with a poor short-term prognosis” | “diseases with predictable poor short-term prognosis” | Life-limiting illness | Severe frailty  Cancer with poor prognosis – ovary, stomach, lung, oesophagus, liver, brain, pancreas, mesothelioma or any secondary  Major chronic illness – pulmonary hypertension, cirrhosis, portal hypertension, dementia.  Or hospital admission with heart failure, COPD or PD  CCI >5 |
|  | “likely unsuitability of the patient for compliance with trial requirements because of psychological or logistical problems” | “predictable psychological or logistical difficulties affecting compliance with the trial requirements” | Psychological or logistical difficulties | Dementia  Care home resident |
|  | “clinical cardiovascular indications [for the use of aspirin] (previous myocardial infarction, unstable angina, stroke, transient ischaemic attacks or vascular bypass” | “… (history of vascular events or diseases)” | History of:  MI  Unstable Angina  Ischaemic stroke  TIA  Vascular disease | Not implemented  Not implemented  Not implemented  Not implemented  Not implemented |
|  | “chronic use of aspirin or other antiplatelet drugs” | “treatment with antiplatelet drugs…” | Prescribed:  Aspirin  Other antiplatelet | Not implemented  Not implemented |
|  | “clinical contraindications [for the use of aspirin] (haemorrhagic syndromes, history of peptic ulcer, high blood pressure (systolic pressure ≥200 mmHg or diastolic pressure ≥ 115 mmHg) uncontrolled by antihypertensive treatment” | “contra-indications to aspirin” | Aspirin contraindicated due to:  Haemorrhagic syndromes  Peptic ulcer  SBP ≥200mmHg on antihypertensive treatment  DBP ≥115mmHg on antihypertensive treatment | Aspirin contraindicated:  Primary thrombocytopaenia ever  Coagulation defects ever  Bleeding disorders:  Platelets <50  Peptic ulcer ever  (Current thiazide or current beta-blocker or current ACE-inhibitor or current ARB or current calcium channel blocker or current cardiac alpha channel blocker)  AND  (SBP ≥ 200 mmHg  OR  DBP ≥115 mmHg) |
|  | “known intolerance to aspirin” |  | Aspirin allergy | Not implemented |
|  | “chronic use of anticoagulants or anti-inflammatory agents (steroidal or other)” | “chronic use of anti-inflammatory agents or anticoagulants” | Prescribed:  Steroids  NSAIDs  Anticoagulants | Current steroid  Current NSAID  Current OAC |

*ACE = angiotensin-converting enzyme, ARB = angiotensin II receptor blocker, BMI = body mass index, CCI = Charlson comorbidity index, COPD = chronic obstructive pulmonary disease, DBP = diastolic blood pressure, FH = family history, MI = myocardial infarction, NSAID = non-steroidal anti-inflammatory drug, OAC = oral anticoagulant, SBP = systolic blood pressure, TIA = transient ischaemic attack*

**In all studies, patients with a history of acute coronary syndrome, myocardial infarction, angina, peripheral arterial disease, ischaemic stroke or transient ischaemic attack were excluded at the point of data extraction.*

# Table S8. Eligibility criteria for Women’s Health Study (WHS)

| Trial Register [49] | Baseline paper [50] | Paper [33] | Criterion for matching | Rule for dataset^*^ |
| --- | --- | --- | --- | --- |
| **Inclusion** | **Inclusion** | **Inclusion** | **Inclusion** | **Inclusion** |
| “Healthy women” | “female” | “women” | Women | Women |
| “ health professionals” | “licensed and registered nurses” | “health professionals” | Female registered nurses | IMD top 3 quintiles [41]  Age <75 |
| “45 years and older” | “45 years of age or older” | “45 years of age or older” | Aged ≥45 | Age ≥45 |
| “No previous history of cardiovascular disease or cancer” | “without previous history of CHD, cerebrovascular disease, cancer (except non-melanoma skin cancer), or any serious illness that might preclude participation” | “no history of coronary heart disease, cerebrovascular disease, cancer (except nonmelanoma skin cancer), or other major chronic illness | No history of:  CHD  Cerebrovascular disease  Cancer (except nonmelanoma skin cancer)  Major chronic illness | No history of:  Not implemented  Any ICH ever  Cancer (except non melanoma) ever  Major chronic illness – Pulmonary hypertension, cirrhosis, portal hypertension, dementia ever or  Hospital admission with heart failure, COPD or PD ever |
| “No contraindications to aspirin or vitamin E” |  |  | Aspirin not contraindicated | No aspirin contraindication:  Primary thrombocytopaenia ever  Coagulation defects ever  Hospital admission with peptic ulcer (first position) in last 12 months |
|  | “postmenopausal or had no intention of becoming pregnant” |  | Postmenopausal or no intention of pregnancy | Not implemented |
|  | “without reported history of serious side effects to any study treatment” | “no history of side effects to any of the study medications” | No allergy to:  Aspirin  Vitamin E | Not implemented |
|  | “not currently taking aspirin, aspirin-containing medications, or NSAID more than once a week or were willing to forego the use of these medications” | “not takin aspirin or nonsteroidal anti-inflammatory medications (NSAIDs) more than once a week (or were willing to forego their use during the trial)” | Not prescribed (more than once a week):  Aspirin  NSAIDs | Not implemented |
|  | “not currently taking anticoagulants or corticosteroids” | “not taking anticoagulants or corticosteroids” | Not prescribed:  Anticoagulants  Steroids | Not taking:  Current OAC  Current steroids |
|  | “not taking supplements of vitamin A or E or beta-carotene more than once a week” | “not taking individual supplements of vitamin A, E or beta carotene more than once a week” | Not prescribed individual supplements of (more than once a week):  Vitamin A  Vitamin E  Beta carotene | Not implemented |
|  | “took at least two thirds of the study medications [during run-in phase]” | “likely to be compliant with long-term treatment” | Likely to be compliant (more than two thirds of medications) | Not implemented |
| **Exclusion** | **Exclusion** | **Exclusion** | **Exclusion** | **Exclusion** |
| None | None | None | None |  |

*CHD = coronary heart disease, COPD = chronic obstructive pulmonary disease, ICH = intracranial haemorrhage, IMD = index of multiple deprivation, NSAID = non-steroidal anti-inflammatory drug, PD = Parkinson’s disease*

**In all studies, patients with a history of acute coronary syndrome, myocardial infarction, angina, peripheral arterial disease, ischaemic stroke or transient ischaemic attack were excluded at the point of data extraction.*

# Table S9. Eligibility criteria for Prevention Of Progression of Arterial Disease And Diabetes (POPADAD)

| Trial Register [51] | Protocol, Baseline or Preliminary Results Paper | Paper [34] | Criterion for matching | Rule for dataset^*^ |
| --- | --- | --- | --- | --- |
| **Inclusion** | **Inclusion** | **Inclusion** | **Inclusion** | **Inclusion** |
| “both Insulin-Dependent Diabetes Mellitus (IDDM) and Non Insulin Dependent Diabetes Mellitus (NIDDM) patients…” | N/A | “type 1 or type 2 diabetes” | Diabetes mellitus | Diabetes mellitus |
| “…of either sex, 40+ years of age” |  | “adults of either sex, aged 40 or more” | Aged ≥40 | Age ≥40 |
| “ankle brachial pressure index (ABPI) of less than 0.98” |  | “asymptomatic peripheral arterial disease as detected by a lower than normal ankle brachial pressure index (≤ 0.99)” | Asymptomatic peripheral arterial disease (ABPI ≤ 0.99) | Not implemented |
| **Exclusion** | **Exclusion** | **Exclusion** | **Exclusion** | **Exclusion** |
| “Participants taking regular aspirin, antioxidant therapy” | N/A | “use aspirin or antioxidant therapy on a regular basis” | Prescribed regular:  Aspirin  Antioxidant therapy | Not implemented |
| “Evidence of symptomatic vascular disease” |  |  | Symptomatic vascular disease | Peripheral vascular disease ever  Intermittent claudication ever |
|  |  | “evidence of symptomatic cardiovascular disease” | Symptomatic CVD | Not implemented |
| “Serious physical illness such as cancer which may curtail life expectancy” |  | “suspected serious physical illness (such as cancer), which might have been expected to curtail life expectancy” | Life-limiting illness | Severe frailty  Cancer with poor prognosis – ovary, stomach, lung, oesophagus, liver, brain, pancreas, mesothelioma or any secondary  Major chronic illness – pulmonary hypertension, cirrhosis, portal hypertension, dementia.  Hospital admission with heart failure, COPD or PD  CCI >5 |
| “Psychiatric illness (reported by GP)” |  | “psychiatric illness (reported by their general practitioner)” | Psychiatric illness | Serious psychiatric illness ever (schizophrenia and BPD) |
| “Congenital heart disease” |  | “congenital heart disease” | Congenital heart disease | Congenital heart disease ever |
| “Pregnancy” |  |  | Pregnant | Not implemented |
|  |  | “peptic ulceration” | Peptic ulcer | Peptic ulcer ever |
|  |  | “severe dyspepsia” | Severe dyspepsia | Dyspepsia ever |
|  |  | “bleeding disorder” | Bleeding disorder | Bleeding disorders ever |
|  |  | “intolerance to aspirin” | Allergy to aspirin | Not implemented |
|  |  | “unable to give informed consent” | Unable to give informed consent | Dementia ever (Duplicate) |

*ABPI = ankle brachial pressure index, BPD = bipolar disorder, CCI = Charlson comorbidity index, COPD = chronic obstructive pulmonary disease, CVD = cardiovascular disease, GP = general practitioner, PD = Parkinson’s disease*

**In all studies, patients with a history of acute coronary syndrome, myocardial infarction, angina, peripheral arterial disease, ischaemic stroke or transient ischaemic attack were excluded at the point of data extraction.*

# Table S10. Eligibility criteria for Japanese Primary prevention of Atherosclerosis with aspirin for Diabetes (JPAD)

| Trial Register [52] | Protocol, Baseline or Preliminary Results Paper | Paper [35] | Criterion for matching | Rule for dataset^*^ |
| --- | --- | --- | --- | --- |
| **Inclusion** | **Inclusion** | **Inclusion** | **Inclusion** | **Inclusion** |
| “type 2 diabetes mellitus…” |  | “diagnosis of type 2 diabetes mellitus” | Diagnosis of type 2 diabetes mellitus | Type 2 diabetes mellitus ever |
| “30 or more years old and 85 years old or less” | N/A | “age between 30 and 85 years” | Aged 30-85 | Age 30-85 |
| “give their informed consent to participate” |  | “ability to provide informed consent” | Able to give informed consent | Absence of dementia ever |
| **Exclusion** | **Exclusion** | **Exclusion** | **Exclusion** | **Exclusion** |
| “electro-cardiographic changes, including ST-segment depression, ST-segment elevation or pathologic Q waves” | N/A | “electro-cardiographic changes consisting of ischemic ST-segment depression, ST-segment elevation, or pathologic Q waves” | Ischaemic ECG changes (ST elevation/depression or pathologic Q waves) | Not implemented |
| “fixed ischemic heart disease, utilizing coronary angiography” |  | “a history of coronary heart disease confirmed by coronary angiography” | Coronary heart disease diagnosed with angiography | Not implemented |
| “cerebral vascular disease, including cerebral infarction, past haemorrhage, and experience of transient ischemic attack” |  | “a history of cerebrovascular disease consisting of cerebral infarction, cerebral haemorrhage, sub-arachnoid haemorrhage, and transient ischemic attack” | History of cerebrovascular disease, consisting of:  Cerebral infarction  Cerebral haemorrhage  Sub-arachnoid haemorrhage  TIA | Not implemented  Any ICH ever  Not implemented |
| “arteriosclerotic disease, which needs internal medicine and/or surgical medical treatment” |  | “a history of arteriosclerotic disease necessitating medical treatment” | Arteriosclerotic disease requiring medical treatment | Not implemented |
| “already taken the following anti-platelet or anti-thrombotic medicine: aspirin, ticlopidine, cilostazol, dipyridamole, trapidil, warfarin, and argatroban” |  | “use of antiplatelet or antithrombotic therapy, defined as aspirin, ticlopidine, cilostazol, dipyridamole, trapidil, warfarin and argatroban” | Prescribed:  Aspirin  Ticlopidine  Cilostazol  Dipyridamole  Trapdil  Warfarin  Argatroban | Current OAC |
| “severe gastric and/or duodenal ulcer” |  | “a history of severe gastric or duodenal ulcer” | Severe gastric or duodenal ulcer | Hospital admission with GI ulcer ever |
| “severe liver dysfunction” |  | “severe liver dysfunction” | Severe liver disease | Severe liver disease:  Cirrhosis ever  Portal hypertension ever  Variceal bleed ever |
| “severe renal dysfunction” |  | “severe renal dysfunction” | Severe renal disease | Severe renal disease:  CKD 4 ever  CKD 5 ever  ESRD ever |
| “allergy for aspirin” |  | “allergy to aspirin” | Allergy to aspirin | Not implemented |
| “atrial fibrillation” |  | “atrial fibrillation” | AF | AF ever |
| “pregnancy or the possible case of pregnancy” |  | “pregnancy” | Pregnant | Not implemented |

*AF = atrial fibrillation, CKD = chronic kidney disease, ECG = electrocardiogram, ESRD = end stage renal disease, GI = gastrointestinal, OAC = oral anticoagulant, TIA = transient ischaemic attack*

**In all studies, patients with a history of acute coronary syndrome, myocardial infarction, angina, peripheral arterial disease, ischaemic stroke or transient ischaemic attack were excluded at the point of data extraction.*

# Table S11. Eligibility criteria for Aspirin for Asymptomatic Atherosclerosis (AAA)

| Trial Register [53] | Protocol, Baseline or Preliminary Results Paper | Paper [36] | Criterion for matching | Rule for dataset^*^ |
| --- | --- | --- | --- | --- |
| **Inclusion** | **Inclusion** | **Inclusion** | **Inclusion** | **Inclusion** |
| “Men and women aged between 50 and 80 years” | N/A | “men and women aged 50 to 75 at baseline…” | Aged 50-75 | Age 50-75 |
| “Ankle brachial pressure index 0.95 or less in at least one limb” |  |  | ABPI ≤ 0.95 in at least one limb | Not implemented |
| “Living in central Scotland (Lothian, Greater Glasgow and Lanarkshire)” |  |  | Living in central Scotland | Not implemented |
| “No history of clinical cardiovascular disease” |  | “…no history of vascular disease”  “not had a heart attack or stroke” | No history of:  CVD, such as MI  Vascular disease  Stroke | No history of:  Not implemented  Not implemented  Any ICH ever |
|  |  | “not taking aspirin or warfarin” | N/A – See exclusion criteria |  |
| **Exclusion** | **Exclusion** | **Exclusion** | **Exclusion** | **Exclusion** |
| “Receiving aspirin and/or other anticoagulants” | N/A | None | Prescribed:  Aspirin  Warfarin  Other anticoagulants | Current OAC |
| “Contraindication to aspirin therapy” |  |  | Aspirin contraindicated | Any aspirin contraindication:  Primary thrombocytopaenia ever  Coagulation defects ever  Hospital admission with peptic ulcer (first position) in last 12 months |

*ABPI = ankle brachial pressure index, CVD = cardiovascular disease, ICH = intracranial haemorrhage, MI = myocardial infarction, OAC = oral anticoagulant*

**In all studies, patients with a history of acute coronary syndrome, myocardial infarction, angina, peripheral arterial disease, ischaemic stroke or transient ischaemic attack were excluded at the point of data extraction.*

# Table S12. Eligibility criteria for Japanese Primary Prevention Project (JPPP)

| Trial Register [54] | Protocol, Baseline or Preliminary Results Paper | Paper [6] | Criterion for matching | Rule for dataset |
| --- | --- | --- | --- | --- |
| **Inclusion** | **Inclusion** | **Inclusion** | **Inclusion** | **Inclusion** |
| **“Elderly patients not previously diagnosed to have any arteriosclerotic disease, including coronary artery disease or cerebrovascular disease, but meeting any one or more of the following criteria (or on medication for any one or more of the following conditions)”:** | N/A |  | **Satisfies at least one of the following inclusion criteria (in italics)** |  |
| “Hypertension: SBP ≥ 140 mmHg or DBP ≥ 90 mmHg” |  | “met Japanese guideline criteria for hypertension (systolic blood pressure [SBP] ≥140 mm Hg or diastolic blood pressure [DBP] ≥ 90 mm Hg)…” | Hypertension, defined as SBP ≥140 mmHg or DBP ≥90 mmHg | Hypertension ever  SBP ≥140  DBP ≥90 |
| “Hyperlipidemia: Hyper-cholesterolemia (total cholesterol ≥ 220 mg/dL or LDL cholesterol ≥ 140 mg/dL) or Hyper-triglyceridemia (Triglycerides ≥ 150 mg/dL) or Low-HDL cholesterolemia (HDL cholesterol < 40 mg/dL)” |  | “…dyslipidemia (total cholesterol ≥ 220 mg/dL or low-density lipoprotein [LDL] cholesterol ≥ 140 mg/dL or high-density lipoprotein [HDL] cholesterol <40mg/dL or triglycerides ≥150 mg/dL…” | Hyper-cholesterolaemia, defined as total cholesterol ≥ 220 mg/dl, LDL ≥140mg/dl, triglycerides ≥150 mg/dl or HDL <40mg/dl | Total cholesterol ≥5.69mmol/l  LDL ≥3.62 mmol/l  Triglycerides ≥3.88mmol/l  HDL <1.03mmol/l  Current lipid lowering drug |
| “Diabetes: Fasting morning blood glucose ≥ 126 mg/dL or casual blood glucose ≥ 200 mg/dL or blood glucose at 2 hrs in the 75-g glucose tolerance test ≥ 200 mg/dL, or HbA1c ≥ 6.5%” |  | “…diabetes mellitus (fasting morning blood glucose ≥ 126 mg/dL or any blood glucose ≥ 200 mg/dl or 2-hour blood glucose ≥ 200 mg/dL in the 75-g glucose tolerance test, or glycated haemoglobin ≥ 6.5%” | Diabetes mellitus, defined as fasting blood glucose ≥126mg/dl, random blood glucose ≥200mg/dl, 2-hour blood glucose ≥200mg/dl in glucose tolerance test or HbA1c ≥6.5% | Diabetes mellitus ever |
| “Age: 60 to 85 years” |  | “aged 60 to 85 years” | Age 60-85 | Age 60-85 |
| “Patients who can give written consent for participation in the study” |  |  | Can give written consent | No history of:  Dementia ever |
|  |  | “had not been diagnosed with atherosclerotic disease” | No diagnosis of atherosclerotic disease | Not implemented |
| **Exclusion** | **Exclusion** | **Exclusion** | **Exclusion** | **Exclusion** |
| “Patients with a history of coronary artery disease or cerebrovascular disease (including transient ischemic attack)” | N/A | “a history of coronary artery disease (including transient ischemic attack [TIA])” | History of:  Coronary artery disease  Cerebrovascular disease (including TIA) | (Duplicated in part)  Any ICH ever |
| “Patients with arteriosclerotic disease requiring surgery or intervention” |  | “atherosclerotic disease requiring surgery or intervention” | Atherosclerotic disease requiring surgical intervention | (Duplicate) |
| “Patients who have or may have atrial fibrillation” |  | “atrial fibrillation (confirmed or suspected)” | Confirmed or suspected AF | AF ever |
| “Patients being treated with aspirin, other antiplatelet agents or anticoagulants” |  | “patients who were receiving antiplatelet agents, anticoagulants… | Prescribed:  Aspirin  Antiplatelets  Anticoagulants | Current OAC |
| “Patients using NSAIDs chronically” |  | “… long-term treatments with non-steroidal anti-inflammatory drugs” | Regularly prescribed NSAIDs | Current NSAID |
| “Patients with a history of hypersensitivity to aspirin or salicylic acid” |  | “…a history of hypersensitivity to aspirin or salicylic acid” | Allergy to aspirin | Not implemented |
| “Patients with peptic ulcers” |  | “Patients with peptic ulcer…” | Peptic ulcer | Peptic ulcer ever |
| “Patients with a bleeding tendency” |  | “…conditions associated with bleeding (eg, von Willebrand disease)…” | Bleeding disorder, such as von Willebrand disease | Bleeding disorders ever |
| “Patients with serious blood abnormalities” |  | “…serious blood abnormalities (eg, clotting factor deficiencies)” | Blood abnormalities, such as clotting factor deficiencies | Clotting disorders ever |
| “Patients with aspirin-sensitive asthma or a history of the same” |  | “patients with aspirin-sensitive asthma…” | Asthma sensitive to aspirin | Not implemented |
| “Patients who are otherwise judged by the investigator to be unsuitable for enrolment in the study” |  |  | Otherwise judged to be unsuitable | Not implemented |

*AF = atrial fibrillation, DBP = diastolic blood pressure, HDL = high density lipoprotein, ICH = intracranial haemorrhage, LDL = low density lipoprotein, NSAID = non-steroidal anti-inflammatory drug, OAC = oral anticoagulant, SBP = systolic blood pressure, TIA = transient ischaemic attack*

**In all studies, patients with a history of acute coronary syndrome, myocardial infarction, angina, peripheral arterial disease, ischaemic stroke or transient ischaemic attack were excluded at the point of data extraction.*

# Table S13. Eligibility criteria for Aspirin to Reduce the Risks of Initial Vascular Events (ARRIVE)

| Trial Register [55] | Protocol, Baseline or Preliminary Results Paper | Paper [13] | Criterion for matching | Rule for dataset^*^ |
| --- | --- | --- | --- | --- |
| **Inclusion** | **Inclusion** | **Inclusion** | **Inclusion** | **Inclusion** |
| **“Males aged 55 years and above with 2 to 4 risk factors”:** | N/A | **“male patients were aged 55 years and older and had between two and four risk factors”:** | **Male, age ≥55 and satisfies 2-4 of the following inclusion criteria (in italics)** | **Men ≥ 55 years old with 2-4 of the following** |
| “Elevated cholesterol (Tchol>200 mg/dL or LDL>130 mg/dL; as measured at screening) irrespective of current treatment” |  | “high cholesterol (total cholesterol >200 mg/dL [5·180 mmol/L] or LDL >130 mg/dL [3·367 mmol/L] for men… irrespective of current treatment” | High cholesterol, defined as total cholesterol >200mg/dl (5.18mmol/l), or LDL >130mg/dl (3.367mmol/l) | Total cholesterol >5.18 mmol/l  LDL cholesterol >3.367 mmol/l |
| “Current smoking: defined as any cigarette smoking in the past 12 months” |  | “current smoking (any cigarette smoking in the past 12 months)” | Smoker (any cigarette in the past 12 months) | Current smoker |
| “Low HDL cholesterol (HDL<40 mg/dL; as measured at screening)” |  | “low HDL cholesterol (<40 mg/dL)” | Low HDL cholesterol, defined as <40mg/dl | HDL cholesterol < 1.03 mmol/l |
| “Elevated blood pressure (SBP>140 mmHg; as measured at screening)” |  | “high blood pressure (systolic blood pressure >140 mm Hg)” | SBP >140mmHg | SBP >140 mmHg |
| “Currently on any medication to treat high blood pressure” |  | “receiving medication to treat high blood pressure” | Prescribed antihypertensive medication | Current thiazide, Beta-blocker, ACE-inhibitor, ARB, calcium channel blocker, Cardiac alpha-blocker |
| “Positive family history of early CHD (a first-degree relative [father, mother, brother, sister, son, daughter] suffered a heart attack [myocardial infarction] before the age of 60 years)” |  | “positive family history of cardiovascular heart disease” | Family history of cardiovascular heart disease, defined as a first-degree relative suffering an MI age <60 | FH: CVD all codes |
| **“Females aged 60 and above with 3 or more risk factors”:** |  | **Females aged 60 and above with 3 or more risk factors:** | **Female, age ≥60 and satisfies 3 or more of the following inclusion criteria (in italics)** | **Female ≥ 60 years old with ≥3 of the following** |
| “Elevated cholesterol (Tchol>240 mg/dL or LDL>160 mg/dL; as measured at screening) irrespective of current treatment” |  | “high cholesterol (total cholesterol >240 mg/dL [6·126 mmol/L] or LDL >160 mg/dL [4·144 mmol/L] for women) irrespective of current treatment” | High cholesterol, defined as total cholesterol >240mg/dl (6.126mmol/l), or LDL >160mg/dl (4.144mmol/l) | Total cholesterol >6.126 mmol/l  LDL cholesterol >3.367 mmol/l |
| “Current smoking: defined as any cigarette smoking in the past 12 months” |  | “current smoking (any cigarette smoking in the past 12 months)” | Smoker (any cigarette in the past 12 months) | Current smoker |
| “Low HDL cholesterol (HDL<40 mg/dL; as measured at screening)” |  | “low HDL cholesterol (<40 mg/dL)” | Low HDL cholesterol, defined as <40mg/dl | HDL cholesterol < 1.03 mmol/l |
| “Elevated blood pressure (SBP>140 mmHg; as measured at screening)” |  | “high blood pressure (systolic blood pressure >140 mm Hg)” | SBP >140mmHg | SBP >140 mmHg |
| “Currently on any medication to treat high blood pressure” |  | “receiving medication to treat high blood pressure” | Prescribed antihypertensive medication | Current thiazide, Beta-blocker, ACE-inhibitor, ARB, calcium channel blocker, Cardiac alpha-blocker |
| “Positive family history of early CHD (a first-degree relative [father, mother, brother, sister, son, daughter] suffered a heart attack [myocardial infarction] before the age of 60 years)” |  | “positive family history of cardiovascular heart disease” | Family history of cardiovascular heart disease, defined as a first-degree relative suffering an MI age <60 | FH: CVD all codes |
| “An understanding and willingness to comply with trial procedures and has given written informed consent to participate in the trial” |  |  | Able to provide informed consent | Dementia ever |
| **Exclusion** | **Exclusion** | **Exclusion** | **Exclusion** | **Exclusion** |
| “History of a documented vascular event, such as MI, stroke, coronary artery angioplasty or stenting, coronary artery bypass graft, relevant arrhythmias, or congestive heart failure or vascular intervention” | N/A | “history of a vascular event, such as stroke, myocardial infarction, coronary artery angioplasty or stenting, coronary artery bypass graft, relevant arrhythmias, congestive heart failure, or vascular intervention” | History of;  Vascular event, such as stroke  MI  Coronary artery angioplasty or stenting  CABG  Arrhythmias  Congestive heart failure  Vascular intervention | Any ICH ever  Not implemented  Not implemented  Not implemented  Arrhythmias ever  Heart failure ever  Not implemented |
| “Patients who are at higher than moderate risk on the basis of their diabetes status, other factors known to the investigator, or the currently used national risk score” |  | “patients with diabetes” | Diabetes | Diabetes mellitus ever |
| “Known contraindications to the study drug, e.g. hypersensitivity to acetylsalicylic acid” |  |  | Aspirin allergy or contraindicated | Any aspirin contraindication:  Primary thrombocytopaenia ever  Coagulation defects ever  Hospital admission with peptic ulcer (first position) in last 12 months |
| “Recent (in the past year) history of gastrointestinal or genitourinary bleeding or other bleeding disorders” |  | “at high risk of gastrointestinal and other bleeding, including those with a history of gastric or duodenal ulcers or gastrointestinal bleeding” | High risk of bleeding  History of:  Gastric/duodenal ulcers  GI bleeding  Genitourinary bleeding | Gastric/duodenal ulcer ever  Hospital admission with any bleeding event ever |
| “Active diagnosed and documented reflux esophagitis” |  |  | Active reflux oesophagitis | Dyspepsia ever |
| “Patients presenting with any medical condition, or psychiatric or substance abuse disorder, that, in the opinion of the investigator, is likely to affect the patient's ability to complete the study or precludes the patient's participation in the study” |  |  | Chronic medical condition  Psychiatric condition  Active substance abuse disorder | Not implemented  Serious psychiatric illness ever (schizophrenia and BPD)  Not implemented |
| “Lactating women or women of childbearing potential” |  |  | Lactating women or women of childbearing potential | Not implemented |
| “Severe liver disease or damage based on the clinical judgment of the investigator” |  |  | Severe liver disease | Severe liver disease:  Cirrhosis ever  Portal hypertension ever  Variceal bleed ever |
| “Severe renal disease or damage based on the clinical judgement of the investigator” |  |  | Severe renal disease | Severe renal disease:  CKD 4 ever  CKD 5 ever  ESRD ever |
| “A definite indication for acetylsalicylic acid therapy, other antiplatelet drug, or anticoagulant in the opinion of the physician” |  | “…required antiplatelet therapy” | Indication for:  Aspirin  Antiplatelets  Anticoagulants | (Duplicate in part, see above)  Not implemented  Not implemented  Current OAC |
| “A history of asthma induced by administration of salicylates or substances with a similar action, notably NSAIDS” |  |  | History of asthma induced by aspirin or NSAIDs | Not implemented |
| “Chronic, frequent (> 5 days/month) use of NSAIDs (including aspirin, or aspirin containing products), COX-2 inhibitors or metamizole” |  | “… frequent use of non-steroidal anti-inflammatory drugs” | Prescribed NSAIDs more than 5 days/month | Regular NSAIDs |
| “Current participation in any other trials involving investigational products within 30 days prior to the Screening Visit” |  |  | Participation in another trial | Not implemented |
| “Current use of an anticoagulant medication” |  |  | Prescribed anticoagulants | See above |
| “Sitting systolic blood pressure greater than 170 mmHg” |  |  | SBP >170mmHg | SBP > 170 mmHg |

*ACE = angiotensin-converting enzyme, ARB = angiotensin II receptor blocker, BPD = bipolar disorder, CHD = coronary heart disease, CKD = chronic kidney disease, CVD = cardiovascular disease, ESRD = end stage renal disease, FH = family history, HDL = high density lipoprotein, ICH = intracranial haemorrhage, LDL = low density lipoprotein, MI = myocardial infarction, NSAID = non-steroidal anti-inflammatory drug, OAC = oral anticoagulant, SBP = systolic blood pressure*

**In all studies, patients with a history of acute coronary syndrome, myocardial infarction, angina, peripheral arterial disease, ischaemic stroke or transient ischaemic attack were excluded at the point of data extraction.*

# Table S14. Eligibility criteria for A Study of Cardiovascular Events iN Diabetes (ASCEND).

| Trial Register [56] | Protocol [12] | Paper [12] | Criterion for matching | Rule for dataset^*^ |
| --- | --- | --- | --- | --- |
| **Inclusion** | **Inclusion** | **Inclusion** | **Inclusion** | **Inclusion** |
| “Males or females with type 1 or type 2 diabetes mellitus” | “Clinical diagnosis of diabetes mellitus: The participant’s own doctor considers them to have type 1 or type 2 diabetes (based on standard WHO or ADA diagnostic criteria)” | “received a diagnosis of diabetes (any type)” | Diagnosis of diabetes mellitus | Diabetes mellitus ever |
| “Aged ≥ 40 years” | “Men or women aged at least 40 years at the time of invitation” | “Men and women at least 40 years of age” | Age ≥40 | Age ≥40 |
| “No previous history of vascular disease” | “No clear indication for aspirin: The participant has no diagnosed occlusive arterial disease (i.e. a history of myocardial infarction, angina pectoris, coronary or non-coronary revascularisation procedure [i.e. peripheral arterial bypass surgery or angioplasty], stroke or transient ischaemic attack)” | “did not have known cardiovascular disease and …” | No indication for aspirin due to a history of:  MI  Angina  Revascularisation procedure  Stroke  TIA | Not implemented  Not implemented  Not implemented  Any ICH ever  Not implemented |
| “No clear contra-indication to aspirin” | “No clear contra-indication to aspirin: The participant is not at high risk of bleeding due to: gastrointestinal haemorrhage or peptic ulcer within the previous 6 months; active hepatic disease such as cirrhosis or active hepatitis; use of warfarin, or other anti-coagulant therapy; or has a history of aspirin allergy” |  | Aspirin not contraindicated, due to:  Active GI bleed or peptic ulceration within last 6 months  Liver disease such as hepatitis or cirrhosis  Warfarin/ anticoagulant use  Allergy to aspirin | Hospital admission with GI bleed in past 6 months  Hospital admission with peptic ulcer in past 6 months  Active liver disease  Current OAC |
| “No other predominant life-threatening medical problem” | “No other predominant life-threatening medical problem: The participant does not have some condition (other than diabetes) that might limit compliance with 5 years of study treatment, such as cancer (other than non-melanoma skin cancer)” |  | No life limiting illness (<5 year prognosis) such as cancer (other than non-melanoma skin cancer) | Severe frailty  Cancer (non-melanoma) in past 5 years Major chronic illness – Pulmonary hypertension, cirrhosis, portal hypertension, dementia  Hospital admission with heart failure, COPD or PD  CCI >5 |
|  | “Substantial uncertainty about whether antiplatelet or omega-3 FA therapy confers worthwhile benefit: Neither the participant nor the participant’s own doctor considers there to be a definite need for the patient to take aspirin or omega-3 FA supplements regularly (or a definite need not to do so)” | “…substantial uncertainty about whether antiplatelet therapy would confer worthwhile benefit” | No indication for antiplatelet therapy | Not implemented |
| **Exclusion** | **Exclusion** | **Exclusion** | **Exclusion** | **Exclusion** |
| “Definite history of myocardial infarction, stroke or arterial revascularisation procedure” |  |  | N/A – See inclusion criteria | N/A – See inclusion criteria |
| “Currently prescribed aspirin, warfarin or any other blood thinning medication” |  |  | Prescribed:  Aspirin | Not implemented |
|  |  | “a clear indication for aspirin” | Indication for aspirin | Duplicate |
|  |  | “a contraindication to aspirin” | N/A – See inclusion criteria | Contraindication to aspirin |
|  |  | “the presence of other clinically significant conditions that might limit adherence to the trial regimen for at least 5 years” | N/A – See inclusion criteria | N/A – See inclusion criteria |

*CCI = Charlson comorbidity index, COPD = chronic obstructive pulmonary disease, GI = gastrointestinal, ICH = intracranial haemorrhage, MI = myocardial infarction, OAC = oral anticoagulant, PD = Parkinson’s disease, TIA = transient ischaemic attack*

**In all studies, patients with a history of acute coronary syndrome, myocardial infarction, angina, peripheral arterial disease, ischaemic stroke or transient ischaemic attack were excluded at the point of data extraction.*

# Table S15. Eligibility criteria for ASPirin for Reducing Events in the Elderly (ASPREE).

| Trial Register [57] | Protocol Paper [7] | Paper [7] | Criterion for matching | Rule for dataset^*^ |
| --- | --- | --- | --- | --- |
| **Inclusion** | **Inclusion** | **Inclusion** | **Inclusion** | **Inclusion** |
| “Men and women” |  |  | **Men and women and satisfies at least one of the following inclusion criteria (in italics)** |  |
| “African American and Hispanic persons age 65 or older” | “US minority (African American and Hispanic) 65 years of age and older” | “… except for U.S. blacks and Hispanics who were aged 65 years and older (no upper age limit)” | Black or Hispanic and aged ≥65 | Black or Hispanic ethnicity  AND  Age ≥65 |
| “Any person from another ethnic minority group and Caucasian persons age 70 or older” | “Non-minority men and women 70 years of age and older” | “aged 70 years and older (no upper age limit)” | Age ≥70 | Age ≥70 |
| “Willing and able to provide informed consent, and willing to accept the study requirements” | “Willing and able to provide informed consent” | “able to give informed consent” | Able to give informed consent | No history of:  Dementia ever |
|  |  | “able to attend a study visit” | Able to attend a study visit | Not current care home resident |
| **Exclusion** | **Exclusion** | **Exclusion** | **Exclusion** | **Exclusion** |
| “A history of a diagnosed cardiovascular event” | “A history of a diagnosed CVD event defined as myocardial infarction (MI), heart failure, angina pectoris, stroke, transient ischemic attack, >50% carotid stenosis or previous carotid endarterectomy or stenting, coronary artery angioplasty or stenting, coronary artery bypass grafting, or abdominal aortic aneurysm” | “a past history of cardiovascular or cerebrovascular event or established CVD, defined as myocardial infarction (MI), heart failure, angina pectoris, stroke, transient ischemic attack, >50% carotid stenosis or previous carotid endarterectomy or stenting, coronary artery angioplasty or stenting, coronary artery bypass grafting, abdominal aortic aneurysm” | History of:  MI  Heart failure  Angina pectoris  Stroke  TIA  >50% carotid stenosis  Carotid endarterectomy  Coronary artery angioplasty or stenting  CABG  AAA | History of:  Not implemented  Heart failure ever  Not implemented  Any ICH ever  Not implemented  Not implemented  Not implemented  Not implemented  Not implemented  AAA ever |
| “A serious intercurrent illness likely to cause death within the next 5 years, such as terminal cancer or obstructive airways disease” | “Serious illness likely to cause death within the next 5 years” | “a condition likely to cause death within 5 years (opinion of the General Practitioner or Primary Care Physician)” | Life limiting illness (<5 year prognosis) | Severe frailty  Cancer with poor prognosis – ovary, stomach, lung, oesophagus, liver, brain, pancreas, mesothelioma or any secondary  Major chronic illness – pulmonary hypertension, cirrhosis, portal hypertension, dementia.  Hospital admission with heart failure, COPD or PD  CCI >5 |
| “A current or recurrent condition with a high risk of major bleeding, ex: cerebral aneurysm” | “A current or recurrent condition with a high risk of major bleeding” | “a condition with a high current or recurrent risk of bleeding…” | High risk of bleeding | Hospital admission with bleeding event ever  Bleeding syndromes |
| “Anemia” | “Anemia (hemoglobin <12 g/dl males, <11 g/dl females)” | “…anemia (hemoglobin <12 g/dl males, <11 g/dl females)” | Anaemia, defined as Hb <12 g/dl in males or <11 g/dl in females | Hb <120 g/l (Men)  Hb <110 g/l (Women) |
| “Absolute contraindication or allergy to aspirin” | “An absolute contraindication or allergy to aspirin” |  | Aspirin allergy or contraindicated | Any aspirin contraindication:  Primary thrombocytopaenia ever  Coagulation defects ever  Hospital admission with peptic ulcer (first position) in last 12 months |
| “Current participation in a clinical trial” | “Current participation in an ongoing clinical trial” | “current participation in another clinical trial” | Participation in another trial | Not implemented |
| “Current continuous use of aspirin or other anti-platelet drug or anticoagulant for secondary prevention. People with previous use of aspirin for primary prevention may enter the trial, provided they agree to cease existing use of aspirin and understand that they may be subsequently randomly allocated to low dose aspirin or placebo” | “Current use of aspirin for secondary prevention”  “Current continuous use of other antiplatelet drug or anticoagulant” | “current continuous use of other antiplatelet or anticoagulant medication”  “current use of aspirin for secondary prevention”  “unwilling to cease regular aspirin being taken for primary prevention” | Prescribed (for secondary prevention):  Aspirin  Antiplatelet  Anticoagulant | Current OAC |
| “A systolic blood pressure ≥180 mmHg and / or a diastolic blood pressure ≥105 mmHg” | “A systolic blood pressure ≥180 mmHg and/or a diastolic blood pressure ≥105mmHg” | “uncontrolled high blood pressure (systolic BP ≥180mmHg and/or diastolic BP ≥105mmHg)” | SBP ≥180mmHg  DBP ≥105mmHg | SBP ≥180mmHg  OR  DBP ≥105mmHg |
| “A history of dementia” | “A history of dementia or a Modified Mini-Mental State Examination (3MS) score ≤77” | “a clinical diagnosis of dementia or score of <78 out of 100 on Modified Mini-Mental State (3MS) examination administered by trained study staff” | Clinical diagnosis of dementia or <78/100 on Modified Mini-Mental State exam | Dementia ever |
| “Severe difficulty or an inability to perform any one of the 6 Katz ADLs” | “Severe difficulty or an inability to perform any one of the 6 Katz activities of daily living (ADLS)” | “physical disability as defined by severe difficulty or inability to perform independently any of the 6 Katz basic activities of daily living (ADLs) which include bathing, transferring from chair or bed, toileting, dressing, eating, walking across a room” | Physical disability, defined as inability to perform any ADLs independently | Duplicate |
| “Non-compliance to taking pill” | “Pill-taking compliance <80% during a 4-week placebo run-in phase” | “pill taking compliance of <80% during a 4-week placebo run-in phase” | Compliance <80% during 4-week run-in | Not implemented |
|  | “A clinical diagnosis of atrial fibrillation” | “a clinical diagnosis of atrial fibrillation” | AF | AF ever |

*AAA = abdominal aortic aneurysm, ADL = activities of daily living, AF = atrial fibrillation, CABG = coronary artery bypass graft, CCI = Charlson comorbidity index, COPD = chronic obstructive pulmonary disease, CVD = cardiovascular disease, DBP = diastolic blood pressure, Hb = haemoglobin, ICH = intracranial haemorrhage, MI = myocardial infarction, OAC = oral anticoagulant, PD = Parkinson’s disease, SBP = systolic blood pressure, TIA = transient ischaemic attack*

**In all studies, patients with a history of acute coronary syndrome, myocardial infarction, angina, peripheral arterial disease, ischaemic stroke or transient ischaemic attack were excluded at the point of data extraction*

# Table S16. Comparisons between trial-eligible in CPRD Gold population, trial-ineligible in CPRD Gold population, and people recruited to trial.

| Trial | Year reported | Proportion ineligible (%) | Mean age ineligible (years) | Mean age eligible (years) | Mean age recruited (years) | Difference in mean age (years)^c^ | | Proportion of ineligible who are women (%) | Proportion of eligible who are women (%) | Proportion of recruited who are women (%) | Difference in proportion women (percentage points)^d^ | |
| --- | --- | --- | --- | --- | --- | --- | --- | --- | --- | --- | --- | --- |
|  |  |  |  |  |  | Ineligible  vs  Eligible | Eligible  vs  Recruited |  |  |  | Ineligible  vs  Eligible | Eligible  vs  Recruited |
| BDT | 1988 | 88.8 | 58.5 | 55.2 | 60.0^a^ | 3.3 | - | 59.4 | 0.0 | 0.0 | 59.4 | N/A |
| PHS | 1989 | 90.6 | 58.5 | 54.4 | 53.2 | 4.1 | -1.2 | 58.1 | 0.0 | 0.0 | 58.1 | N/A |
| ETDRS | 1992 | 98.8 | 58.1 | 57.8 | 50.0^a^ | 0.3 | - | 52.9 | 39.8 | 43.5 | 13.1 | 3.7 |
| TPT | 1998 | 97.2 | 58.0 | 62.1 | 57.5 | -4.1 | -4.6 | 54.2 | 0.0 | 0.0 | 54.2 | N/A |
| HOT | 1998 | 99.4 | 58.1 | 61.3 | 61.5 | -3.2 | 0.2 | 52.7 | 44.5 | 47.0 | 8.2 | 2.5 |
| PPP | 2001 | 65.0 | 53.6 | 66.4 | 64.4 | -12.8 | -2.0 | 51.7 | 54.6 | 57.5 | -2.9 | 2.9 |
| WHS | 2005 | 77.1 | 58.3 | 57.5 | 54.6 | 0.8 | -2.9 | 38.6 | 100.0 | 100.0 | -61.4 | N/A |
| JPAD | 2008 | 94.4 | 57.8 | 63.2 | 64.5 | -5.4 | 1.3 | 53.1 | 45.7 | 45.4 | 7.4 | -0.3 |
| POPADAD | 2008 | 95.3 | 57.9 | 63.1 | 60.1 | -5.2 | -3.0 | 53.2 | 42.9 | 57.1 | 10.3 | 14.2 |
| AAA | 2010 | 42.5 | 54.8 | 60.5 | 62.0 | -5.7 | 1.5 | 53.2 | 52.4 | 71.5 | 0.8 | 19.1 |
| JPPP | 2014 | 75.7 | 54.5 | 69.4 | 70.5 | -14.9 | 1.1 | 51.5 | 56.5 | 57.7 | -5.0 | 1.2 |
| ASPREE | 2018 | 85.7 | 55.0 | 76.7 | 74.0^b^ | -21.7 | - | 51.5 | 59.7 | 56.4 | -8.2 | -3.3 |
| ARRIVE | 2018 | 90.7 | 57.2 | 67.0 | 63.9 | -9.8 | -3.1 | 55.7 | 23.3 | 29.6 | 32.4 | 6.3 |
| ASCEND | 2018 | 93.8 | 57.8 | 63.2 | 63.3 | -5.4 | 0.1 | 53.3 | 44.3 | 37.4 | 9.0 | -6.9 |

*a. Median estimated from published data*

*b. Median reported by trial*

*c. Positive value means ineligible older than eligible, or eligible older than recruited*

*d. Positive value means ineligible more commonly women than eligible, or eligible more commonly women than ineligible*

*N/A – not applicable (three trials only recruit men, one trial only recruits women)*

# Table S17. Differences in characteristics between the trial-eligible and trial-ineligible in each trial.

|  | **% of ineligible**  **(95% CI)** | **% of eligible**  **(95% CI)** | **p value** |
| --- | --- | --- | --- |
| **BDT** | | | |
| Mean age in years (SE) | 58.5 (0.0) | 55.2 (0.0) | <0.001 |
| Proportion women | 59.4 (59.2, 59.5) | 0.0 (0.0, 0.0) | <0.001 |
| Mean BMI in kg/m² (SE) | 27.5 (0.0) | 27.2 (0.0) | 0.394 |
| Minority ethnic group | 5.2 (5.2, 5.3) | 3.3 (3.2, 3.5) | <0.001 |
| Moderate or severe frailty | 8.0 (8.0, 8.1) | 1.6 (1.5, 1.7) | <0.001 |
| Care home resident | 0.5 (0.5, 0.5) | 0.1 (0.1, 0.1) | <0.001 |
| Diabetes | 7.5 (7.4, 7.5) | 5.9 (5.7, 6.0) | <0.001 |
| Hypertension | 26.6 (26.5, 26.8) | 20.3 (20.0, 20.6) | <0.001 |
| Current or ex-smoker | 36.3 (36.2, 36.5) | 31.3 (31.0, 31.7) | <0.001 |
| Statins | 13.5 (13.4, 13.6) | 12.0 (11.7, 12.3) | <0.001 |
| Aspirin | 2.7 (2.7, 2.8) | 1.6 (1.5, 1.7) | <0.001 |
| Oral anticoagulants | 1.8 (1.7, 1.8) | 1.4 (1.3, 1.5) | <0.001 |
| NSAIDs | 3.2 (3.1, 3.2) | 1.7 (1.6, 1.8) | <0.001 |
| SSRIs | 6.6 (6.6, 6.7) | 2.6 (2.5, 2.7) | <0.001 |
| **PHS** | | | |
| Mean age in years (SE) | 58.5 (0.0) | 54.4 (0.0) | <0.001 |
| Proportion women | 58.1 (58.0, 58.3) | 0.0 (0.0, 0.0) | <0.001 |
| Mean BMI in kg/m² (SE) | 27.6 (0.0) | 27.0 (0.0) | <0.001 |
| Minority ethnic group | 5.2 (5.1, 5.2) | 3.4 (3.2, 3.5) | <0.001 |
| Moderate or severe frailty | 8.0 (7.9, 8.0) | 1.1 (1.0, 1.2) | <0.001 |
| Care home resident | 0.5 (0.5, 0.5) | 0.1 (0.1, 0.1) | <0.001 |
| Diabetes | 7.5 (7.5, 7.6) | 4.9 (4.7, 5.1) | <0.001 |
| Hypertension | 26.8 (26.7, 27.0) | 16.9 (16.6, 17.2) | <0.001 |
| Current or ex-smoker | 36.3 (36.1, 36.4) | 30.9 (30.5, 31.3) | <0.001 |
| Statins | 13.7 (13.6, 13.8) | 9.8 (9.6, 10.1) | <0.001 |
| Aspirin | 2.7 (2.7, 2.8) | 1.3 (1.2, 1.4) | <0.001 |
| Oral anticoagulants | 1.8 (1.7, 1.8) | 1.1 (1.0, 1.2) | <0.001 |
| NSAIDs | 3.3 (3.3, 3.4) | 0.0 (0.0, 0.0) | <0.001 |
| SSRIs | 6.6 (6.5, 6.6) | 2.4 (2.3, 2.5) | <0.001 |
| **ETDRS** | | | |
| Mean age in years (SE) | 58.1 (0.0) | 57.8 (0.1) | <0.001 |
| Proportion women | 52.9 (52.7, 53.0) | 39.8 (38.7, 41.0) | <0.001 |
| Mean BMI in kg/m² (SE) | 27.5 (0.0) | 31.3 (0.1) | <0.001 |
| Minority ethnic group | 4.9 (4.9, 5.0) | 10.7 (10.0, 11.5) | <0.001 |
| Moderate or severe frailty | 7.2 (7.1, 7.2) | 20.2 (19.3, 21.1) | <0.001 |
| Care home resident | 0.4 (0.4, 0.5) | 0.2 (0.1, 0.3) | 0.001 |
| Diabetes | 6.1 (6.0, 6.2) | 100.0 (99.9, 100.0) | <0.001 |
| Hypertension | 25.5 (25.4, 25.6) | 60.8 (59.6, 61.9) | <0.001 |
| Current or ex-smoker | 35.7 (35.6, 35.9) | 38.3 (37.2, 39.5) | <0.001 |
| Statins | 12.7 (12.6, 12.8) | 61.8 (60.7, 62.9) | <0.001 |
| Aspirin | 2.4 (2.4, 2.5) | 14.3 (13.5, 15.1) | <0.001 |
| Oral anticoagulants | 1.7 (1.7, 1.8) | 0.0 (0.0, 0.1) | <0.001 |
| NSAIDs | 3.0 (2.9, 3.0) | 4.9 (4.5, 5.5) | <0.001 |
| SSRIs | 6.2 (6.1, 6.2) | 9.0 (8.3, 9.6) | <0.001 |
| **TPT** | | | |
| Mean age in years (SE) | 58.0 (0.0) | 62.1 (0.0) | <0.001 |
| Proportion women | 54.2 (54.1, 54.4) | 0.0 (0.0, 0.0) | <0.001 |
| Mean BMI in kg/m² (SE) | 27.4 (0.0) | 30.4 (0.0) | <0.001 |
| Minority ethnic group | 5.0 (5.0, 5.1) | 3.9 (3.7, 4.3) | <0.001 |
| Moderate or severe frailty | 7.4 (7.4, 7.5) | 3.4 (3.1, 3.7) | <0.001 |
| Care home resident | 0.5 (0.4, 0.5) | 0.1 (0.0, 0.1) | <0.001 |
| Diabetes | 6.7 (6.6, 6.7) | 28.6 (27.9, 29.3) | <0.001 |
| Hypertension | 25.0 (24.9, 25.2) | 56.2 (55.5, 57.0) | <0.001 |
| Current or ex-smoker | 34.7 (34.6, 34.8) | 73.7 (73.0, 74.3) | <0.001 |
| Statins | 12.7 (12.6, 12.7) | 36.6 (35.9, 37.4) | <0.001 |
| Aspirin | 2.5 (2.4, 2.5) | 6.1 (5.7, 6.5) | <0.001 |
| Oral anticoagulants | 1.8 (1.7, 1.8) | 0.0 (0.0, 0.0) | <0.001 |
| NSAIDs | 3.1 (3.0, 3.1) | 0.0 (0.0, 0.0) | <0.001 |
| SSRIs | 6.4 (6.3, 6.4) | 0.0 (0.0, 0.0) | <0.001 |
| **HOT** | | | |
| Mean age in years (SE) | 58.1 (0.0) | 61.3 (0.1) | <0.001 |
| Proportion women | 52.7 (52.6, 52.9) | 44.5 (42.8, 46.2) | <0.001 |
| Mean BMI in kg/m² (SE) | 27.5 (0.0) | 30.1 (0.1) | <0.001 |
| Minority ethnic group | 5.0 (5.0, 5.1) | 6.4 (5.6, 7.3) | <0.001 |
| Moderate or severe frailty | 7.3 (7.3, 7.4) | 6.2 (5.4, 7.1) | <0.001 |
| Care home resident | 0.4 (0.4, 0.5) | 0.1 (0.0, 0.3) | <0.001 |
| Diabetes | 7.3 (7.2, 7.3) | 10.3 (9.3, 11.4) | <0.001 |
| Hypertension | 25.5 (25.4, 25.6) | 100.0 (99.8, 100.0) | <0.001 |
| Current or ex-smoker | 35.8 (35.7, 35.9) | 34.0 (32.3, 35.7) | <0.001 |
| Statins | 13.3 (13.2, 13.4) | 19.7 (18.4, 21.2) | <0.001 |
| Aspirin | 2.6 (2.5, 2.6) | 3.1 (2.6, 3.8) | <0.001 |
| Oral anticoagulants | 1.7 (1.7, 1.8) | 0.0 (0.0, 0.2) | <0.001 |
| NSAIDs | 3.0 (3.0, 3.0) | 3.4 (2.8, 4.1) | <0.001 |
| SSRIs | 6.2 (6.1, 6.3) | 5.0 (4.3, 5.9) | <0.001 |
| **PPP** | | | |
| Mean age in years (SE) | 53.6 (0.0) | 66.4 (0.0) | <0.001 |
| Proportion women | 51.7 (51.5, 51.8) | 54.6 (54.4, 54.9) | <0.001 |
| Mean BMI in kg/m² (SE) | 26.9 (0.0) | 28.7 (0.0) | <0.001 |
| Minority ethnic group | 5.6 (5.5, 5.7) | 3.9 (3.8, 4.0) | <0.001 |
| Moderate or severe frailty | 7.3 (7.2, 7.4) | 7.3 (7.2, 7.4) | 0.759 |
| Care home resident | 0.7 (0.7, 0.7) | 0.0 (0.0, 0.0) | <0.001 |
| Diabetes | 4.8 (4.7, 4.9) | 11.9 (11.8, 12.1) | <0.001 |
| Hypertension | 16.5 (16.3, 16.6) | 43.5 (43.2, 43.7) | <0.001 |
| Current or ex-smoker | 36.7 (36.6, 36.9) | 34.0 (33.8, 34.2) | <0.001 |
| Statins | 7.5 (7.4, 7.6) | 24.1 (23.9, 24.3) | <0.001 |
| Aspirin | 1.5 (1.5, 1.6) | 4.6 (4.5, 4.6) | <0.001 |
| Oral anticoagulants | 2.6 (2.6, 2.7) | 0.0 (0.0, 0.0) | <0.001 |
| NSAIDs | 4.6 (4.6, 4.7) | 0.0 (0.0, 0.0) | <0.001 |
| SSRIs | 6.6 (6.6, 6.7) | 5.3 (5.2, 5.4) | <0.001 |
| **WHS** | | | |
| Mean age in years (SE) | 58.3 (0.0) | 57.5 (0.0) | <0.001 |
| Proportion women | 38.6 (38.5, 38.8) | 100.0 (100.0, 100.0) | <0.001 |
| Mean BMI in kg/m² (SE) | 27.6 (0.0) | 27.1 (0.0) | <0.001 |
| Minority ethnic group | 5.2 (5.1, 5.2) | 4.5 (4.4, 4.6) | <0.001 |
| Moderate or severe frailty | 8.3 (8.3, 8.4) | 3.9 (3.8, 4.0) | <0.001 |
| Care home resident | 0.6 (0.6, 0.6) | 0.0 (0.0, 0.0) | <0.001 |
| Diabetes | 8.1 (8.0, 8.2) | 4.5 (4.4, 4.6) | <0.001 |
| Hypertension | 27.5 (27.4, 27.6) | 20.5 (20.3, 20.8) | <0.001 |
| Current or ex-smoker | 37.6 (37.5, 37.8) | 29.6 (29.3, 29.8) | <0.001 |
| Statins | 14.6 (14.5, 14.7) | 9.2 (9.0, 9.3) | <0.001 |
| Aspirin | 3.0 (2.9, 3.0) | 1.2 (1.1, 1.3) | <0.001 |
| Oral anticoagulants | 2.2 (2.2, 2.3) | 0.0 (0.0, 0.0) | <0.001 |
| NSAIDs | 3.0 (3.0, 3.1) | 3.0 (2.9, 3.1) | 0.477 |
| SSRIs | 5.8 (5.8, 5.9) | 7.4 (7.2, 7.5) | <0.001 |
| **POPADAD** | | | |
| Mean age in years (SE) | 57.9 (0.0) | 63.1 (0.1) | <0.001 |
| Proportion women | 53.2 (53.0, 53.3) | 42.9 (42.4, 43.5) | <0.001 |
| Mean BMI in kg/m² (SE) | 27.3 (0.0) | 31.1 (0.0) | <0.001 |
| Minority ethnic group | 4.8 (4.8, 4.9) | 8.7 (8.4, 9.1) | <0.001 |
| Moderate or severe frailty | 6.7 (6.6, 6.8) | 19.8 (19.3, 20.2) | <0.001 |
| Care home resident | 0.5 (0.4, 0.5) | 0.3 (0.2, 0.3) | <0.001 |
| Diabetes | 2.7 (2.7, 2.7) | 100.0 (100.0, 100.0) | <0.001 |
| Hypertension | 24.1 (24.0, 24.2) | 62.0 (61.4, 62.6) | <0.001 |
| Current or ex-smoker | 35.7 (35.5, 35.8) | 38.1 (37.5, 38.7) | <0.001 |
| Statins | 11.0 (11.0, 11.1) | 59.6 (59.0, 60.2) | <0.001 |
| Aspirin | 2.1 (2.0, 2.1) | 12.9 (12.5, 13.4) | <0.001 |
| Oral anticoagulants | 1.7 (1.6, 1.7) | 2.7 (2.5, 2.9) | <0.001 |
| NSAIDs | 2.9 (2.9, 3.0) | 4.5 (4.3, 4.8) | <0.001 |
| SSRIs | 6.2 (6.1, 6.2) | 6.9 (6.6, 7.3) | <0.001 |
| **JPAD** | | | |
| Mean age in years (SE) | 57.8 (0.0) | 63.2 (0.1) | <0.001 |
| Proportion women | 53.1 (53.0, 53.2) | 45.7 (45.1, 46.2) | <0.001 |
| Mean BMI in kg/m² (SE) | 27.2 (0.0) | 31.6 (0.0) | <0.001 |
| Minority ethnic group | 4.7 (4.6, 4.8) | 10.3 (10.0, 10.7) | <0.001 |
| Moderate or severe frailty | 6.2 (6.2, 6.3) | 25.9 (25.4, 26.4) | <0.001 |
| Care home resident | 0.5 (0.4, 0.5) | 0.3 (0.3, 0.4) | 0.002 |
| Diabetes | 1.8 (1.8, 1.9) | 100.0 (100.0, 100.0) | <0.001 |
| Hypertension | 23.7 (23.6, 23.8) | 63.9 (63.3, 64.4) | <0.001 |
| Current or ex-smoker | 35.6 (35.4, 35.7) | 39.1 (38.6, 39.7) | <0.001 |
| Statins | 10.5 (10.4, 10.6) | 61.5 (61.0, 62.0) | <0.001 |
| Aspirin | 2.0 (1.9, 2.0) | 12.9 (12.5, 13.2) | <0.001 |
| Oral anticoagulants | 1.8 (1.8, 1.8) | 0.0 (0.0, 0.0) | <0.001 |
| NSAIDs | 2.9 (2.8, 2.9) | 5.2 (5.0, 5.5) | <0.001 |
| SSRIs | 6.0 (6.0, 6.1) | 8.6 (8.3, 8.9) | <0.001 |
| **AAA** | | | |
| Mean age in years (SE) | 54.8 (0.0) | 60.5 (0.0) | <0.001 |
| Proportion women | 53.2 (53.0, 53.4) | 52.4 (52.2, 52.5) | <0.001 |
| Mean BMI in kg/m² (SE) | 27.2 (0.0) | 27.8 (0.0) | <0.001 |
| Minority ethnic group | 6.0 (5.9, 6.1) | 4.3 (4.2, 4.3) | <0.001 |
| Moderate or severe frailty | 9.7 (9.6, 9.8) | 5.6 (5.5, 5.6) | <0.001 |
| Care home resident | 0.7 (0.7, 0.8) | 0.2 (0.2, 0.3) | <0.001 |
| Diabetes | 6.2 (6.1, 6.3) | 8.1 (8.0, 8.2) | <0.001 |
| Hypertension | 23.0 (22.9, 23.2) | 28.0 (27.9, 28.2) | <0.001 |
| Current or ex-smoker | 36.0 (35.8, 36.2) | 35.6 (35.4, 35.8) | 0.001 |
| Statins | 10.1 (10.0, 10.2) | 15.7 (15.6, 15.9) | <0.001 |
| Aspirin | 2.7 (2.7, 2.8) | 2.5 (2.4, 2.5) | <0.001 |
| Oral anticoagulants | 4.0 (3.9, 4.1) | 0.0 (0.0, 0.0) | <0.001 |
| NSAIDs | 2.2 (2.2, 2.3) | 3.6 (3.5, 3.7) | <0.001 |
| SSRIs | 6.3 (6.2, 6.4) | 6.1 (6.0, 6.2) | 0.013 |
| **JPPP** | | | |
| Mean age in years (SE) | 54.5 (0.0) | 69.4 (0.0) | <0.001 |
| Proportion women | 51.5 (51.3, 51.6) | 56.5 (56.2, 56.7) | <0.001 |
| Mean BMI in kg/m² (SE) | 27.4 (0.0) | 27.8 (0.0) | <0.001 |
| Minority ethnic group | 5.6 (5.5, 5.7) | 3.3 (3.2, 3.4) | <0.001 |
| Moderate or severe frailty | 6.4 (6.3, 6.4) | 10.3 (10.1, 10.4) | <0.001 |
| Care home resident | 0.5 (0.5, 0.5) | 0.2 (0.2, 0.3) | <0.001 |
| Diabetes | 5.4 (5.4, 5.5) | 13.1 (12.9, 13.3) | <0.001 |
| Hypertension | 18.2 (18.1, 18.3) | 49.8 (49.6, 50.1) | <0.001 |
| Current or ex-smoker | 36.0 (35.9, 36.1) | 35.1 (34.8, 35.3) | <0.001 |
| Statins | 8.1 (8.0, 8.2) | 29.7 (29.5, 29.9) | <0.001 |
| Aspirin | 1.7 (1.6, 1.7) | 5.4 (5.3, 5.6) | <0.001 |
| Oral anticoagulants | 2.3 (2.2, 2.3) | 0.0 (0.0, 0.0) | <0.001 |
| NSAIDs | 2.5 (2.4, 2.5) | 4.6 (4.5, 4.7) | <0.001 |
| SSRIs | 6.6 (6.5, 6.6) | 5.0 (4.9, 5.1) | <0.001 |
| **ARRIVE** | | | |
| Mean age in years (SE) | 57.2 (0.0) | 67.0 (0.0) | <0.001 |
| Proportion women | 55.7 (55.6, 55.9) | 23.3 (22.9, 23.6) | <0.001 |
| Mean BMI in kg/m² (SE) | 27.5 (0.0) | 27.9 (0.0) | <0.001 |
| Minority ethnic group | 5.3 (5.2, 5.4) | 2.3 (2.2, 2.5) | <0.001 |
| Moderate or severe frailty | 7.7 (7.6, 7.7) | 4.0 (3.8, 4.2) | <0.001 |
| Care home resident | 0.5 (0.5, 0.5) | 0.2 (0.1, 0.2) | <0.001 |
| Diabetes | 8.0 (8.0, 8.1) | 0.0 (0.0, 0.0) | <0.001 |
| Hypertension | 23.3 (23.2, 23.5) | 51.0 (50.6, 51.4) | <0.001 |
| Current or ex-smoker | 33.6 (33.5, 33.8) | 56.5 (56.1, 57.0) | <0.001 |
| Statins | 12.6 (12.5, 12.6) | 20.9 (20.5, 21.2) | <0.001 |
| Aspirin | 2.5 (2.4, 2.5) | 3.5 (3.3, 3.6) | <0.001 |
| Oral anticoagulants | 1.9 (1.9, 1.9) | 0.0 (0.0, 0.0) | <0.001 |
| NSAIDs | 3.3 (3.3, 3.4) | 0.0 (0.0, 0.0) | <0.001 |
| SSRIs | 6.5 (6.4, 6.5) | 3.6 (3.5, 3.8) | <0.001 |
| **ASPREE** | | | |
| Mean age in years (SE) | 55.0 (0.0) | 76.7 (0.0) | <0.001 |
| Proportion women | 51.5 (51.4, 51.7) | 59.7 (59.3, 60.0) | <0.001 |
| Mean BMI in kg/m² (SE) | 27.6 (0.0) | 26.9 (0.0) | <0.001 |
| Minority ethnic group | 5.4 (5.3, 5.4) | 2.8 (2.7, 2.9) | <0.001 |
| Moderate or severe frailty | 6.1 (6.1, 6.2) | 14.3 (14.1, 14.6) | <0.001 |
| Care home resident | 0.5 (0.5, 0.5) | 0.0 (0.0, 0.0) | <0.001 |
| Diabetes | 6.7 (6.6, 6.7) | 11.1 (10.9, 11.3) | <0.001 |
| Hypertension | 21.8 (21.6, 21.9) | 50.8 (50.4, 51.1) | <0.001 |
| Current or ex-smoker | 36.4 (36.2, 36.5) | 32.2 (31.9, 32.5) | <0.001 |
| Statins | 10.7 (10.6, 10.8) | 29.3 (28.9, 29.6) | <0.001 |
| Aspirin | 1.9 (1.9, 2.0) | 6.5 (6.3, 6.7) | <0.001 |
| Oral anticoagulants | 2.0 (2.0, 2.0) | 0.0 (0.0, 0.0) | <0.001 |
| NSAIDs | 2.9 (2.8, 2.9) | 3.9 (3.8, 4.1) | <0.001 |
| SSRIs | 6.6 (6.5, 6.7) | 3.8 (3.7, 3.9) | <0.001 |
| **ASCEND** | | | |
| Mean age in years (SE) | 57.8 (0.0) | 63.2 (0.1) | <0.001 |
| Proportion women | 53.3 (53.1, 53.4) | 44.3 (43.7, 44.8) | <0.001 |
| Mean BMI in kg/m² (SE) | 27.3 (0.0) | 31.1 (0.0) | <0.001 |
| Minority ethnic group | 4.7 (4.7, 4.8) | 9.7 (9.4, 10.0) | <0.001 |
| Moderate or severe frailty | 6.2 (6.2, 6.3) | 23.7 (23.2, 24.1) | <0.001 |
| Care home resident | 0.4 (0.4, 0.5) | 0.4 (0.3, 0.4) | 0.015 |
| Diabetes | 1.2 (1.2, 1.2) | 100.0 (100.0, 100.0) | <0.001 |
| Hypertension | 23.5 (23.4, 23.6) | 62.2 (61.7, 62.7) | <0.001 |
| Current or ex-smoker | 35.6 (35.5, 35.7) | 38.6 (38.1, 39.1) | <0.001 |
| Statins | 10.3 (10.2, 10.3) | 60.2 (59.7, 60.7) | <0.001 |
| Aspirin | 1.9 (1.9, 1.9) | 13.1 (12.8, 13.5) | <0.001 |
| Oral anticoagulants | 1.8 (1.8, 1.9) | 0.0 (0.0, 0.0) | <0.001 |
| NSAIDs | 2.9 (2.8, 2.9) | 4.8 (4.6, 5.1) | <0.001 |
| SSRIs | 6.1 (6.0, 6.1) | 8.2 (7.9, 8.5) | <0.001 |

*BMI – body mass index, IQR – interquartile range, NSAID – non-steroidal anti-inflammatory drug, SSRI – selective serotonin reuptake inhibitor*

# Table S18. Hazards of major adverse cardiovascular events and bleeding events in the trial ineligible compared with the trial-eligible

| Trial | MACE  Hazard Ratio (95% CI) | | Bleeding Events  Hazard Ratio (95% CI) | |
| --- | --- | --- | --- | --- |
|  | Unadjusted | Adjusted for age and sex | Unadjusted | Adjusted for age and sex |
| BDT | 1.17  (1.10-1.25) | 1.35  (1.26-1.44) | 1.50  (1.38-1.63) | 1.54  (1.41-1.68) |
| PHS | 1.35  (1.25-1.45) | 1.43  (1.33-1.54) | 1.70  (1.54-1.87) | 1.68  (1.52-1.85) |
| ETDRS | 0.45  (0.40-0.51) | 0.41  (0.37-0.46) | 0.98  (0.80-1.20) | 0.89  (0.72-1.08) |
| HOT | 0.63  (0.51-0.77) | 0.78  (0.64-0.95) | 0.73  (0.57-0.95) | 0.82  (0.63-1.06) |
| TPT | 0.54  (0.50-0.59) | 0.68  (0.62-0.75) | 1.05  (0.91-1.20) | 1.07  (0.93-1.24) |
| PPP | 0.55  (0.53-0.57) | 1.15  (1.11-1.20) | 0.91  (0.87-0.96) | 1.57  (1.49-1.65) |
| WHS | 2.78  (2.61-2.96) | 2.02  (1.88-2.17) | 1.59  (1.49-1.69) | 1.43  (1.33-1.53) |
| POPADAD | 0.47  (0.44-0.51) | 0.67  (0.63-0.71) | 0.86  (0.78-0.95) | 1.06  (0.96-1.17) |
| JPAD | 0.48  (0.45-0.51) | 0.68  (0.64-0.72) | 0.79  (0.73-0.87) | 0.98  (0.89-1.07) |
| AAA | 1.04  (1.00-1.08) | 1.84  (1.75-1.95) | 1.20  (1.15-1.26) | 2.13  (1.98-2.30) |
| JPPP | 0.52  (0.50-0.54) | 1.26  (1.21-1.32) | 0.81  (0.77-0.86) | 1.57  (1.48-1.67) |
| ARRIVE | 0.50  (0.48-0.53) | 0.95  (0.90-1.00) | 0.89  (0.83-0.96) | 1.30  (1.20-1.40) |
| ASPREE | 0.40  (0.38-0.41) | 1.92  (1.82-2.03) | 0.62  (0.59-0.65) | 2.54  (2.36-2.73) |
| ASCEND | 0.45  (0.43-0.48) | 0.64  (0.60-0.67) | 0.83  (0.76-0.91) | 1.02  (0.93-1.11) |

*AAA - Aspirin for Asymptomatic Atherosclerosis, ARRIVE - Use of Aspirin to Reduce Risk of Initial Vascular Events in patients at moderate risk of cardiovascular disease, ASCEND - A Study of Cardiovascular Events iN Diabetes, ASPREE - , BDT – British Doctor’s Trial, CI – confidence interval, ETDRS - Early Treatment Diabetic Retinopathy Study, HOT - Hypertension Optimal Treatment, JPAD - Japanese Primary prevention of Atherosclerosis with aspirin for Diabetes, JPPP - Japanese Primary Prevention Project, MACE – major adverse cardiovascular events, PHS - Physician’s Health Study, POPADAD - Prevention Of Progression of Arterial Disease And Diabetes, PPP - Primary Prevention Project, TPT - Thrombosis Prevention Trial, WHS - Women’s Health Study*

# Table S19. Time varying hazards of non-cardiovascular deaths in the trial ineligible compared with the trial-eligible

| Trial | Unadjusted time-varying HRs for Non-CVD Death | | | | Time varying HRs for Non-CVD Death, adjusted for age and sex | | | |
| --- | --- | --- | --- | --- | --- | --- | --- | --- |
|  | Maximum | | Minimum | | Maximum | | Minimum | |
|  | Hazard ratio (95% CI) | Month of follow-up | Hazard ratio (95% CI) | Month of follow-up | Hazard ratio (95% CI) | Month of follow-up | Hazard ratio (95% CI) | Month of follow-up |
| BDT | 3.39  (2.41-4.78) | 21 | 2.24  (1.44-3.47) | 36 | 2.62  (1.88-3.66) | 24 | 1.78  (1.15-2.75) | 36 |
| PHS | 6.64  (5.53-7.96) | 3 | 2.36  (1.25-4.45) | 36 | 4.51  (3.76-5.41) | 3 | 1.67  (0.89-3.15) | 36 |
| ETDRS | 2.10  (0.86-5.10) | 12 | 1.00  (0.37-2.71) | 36 | 1.23  (0.51-3.00) | 12 | 0.61  (0.23-1.66) | 36 |
| HOT | 3.75  (2.13-6.60) | 3 | 0.97  (0.20-4.61) | 27 | 4.07  (2.31-7.16) | 3 | 1.07  (0.23-5.06) | 27 |
| TPT | 3.12  (2.45-3.98) | 3 | 1.43  (0.62-3.27) | 36 | 1.99  (1.56-2.53) | 3 | 0.95  (0.41-2.18) | 36 |
| PPP | 1.51  (1.43-1.60) | 3 | 0.64  (0.53-0.77) | 36 | 4.20  (3.98-4.44) | 3 | 1.92  (1.58-2.33) | 36 |
| WHS | 11.42  (9.91-13.17) | 3 | 3.68  (2.26-6.01) | 36 | 9.47  (8.22-10.92) | 3 | 3.24  (1.99-5.28) | 36 |
| POPADAD | 0.90  (0.81-1.00) | 3 | 0.64  (0.44-0.93) | 36 | 1.33  (1.19-1.48) | 3 | 0.95  (0.65-1.39) | 36 |
| JPAD | 0.97  (0.87-1.07) | 3 | 0.71  (0.49-1.02) | 36 | 1.44  (1.30-1.60) | 3 | 1.08  (0.75-1.56) | 36 |
| AAA | 2.65  (2.52-2.79) | 3 | 2.20  (1.84-2.65) | 36 | 3.73  (3.20-4.35) | 15 | 3.40  (2.83-4.09) | 36 |
| JPPP | 1.02  (0.96-1.07) | 3 | 0.60  (0.49-0.73) | 36 | 3.38  (3.19-3.57) | 3 | 2.18  (1.79-2.66) | 36 |
| ARRIVE | 1.33  (1.21-1.46) | 3 | 0.73  (0.53-1.00) | 36 | 2.45  (2.23-2.68) | 3 | 1.39  (1.01-1.92) | 36 |
| ASPREE | 0.74  (0.70-0.79) | 3 | 0.29  (0.24-0.36) | 36 | 7.67  (7.21-8.15) | 3 | 3.50  (2.83-4.33) | 36 |
| ASCEND | 0.93  (0.84-1.03) | 3 | 0.67  (0.52-0.87) | 30 | 1.38  (1.26-1.53) | 3 | 1.01  (0.78-1.32) | 30 |

*Month of follow-up was measured quarterly (i.e., month 3 was the first HR measurement and month 36 the last)*

*AAA - Aspirin for Asymptomatic Atherosclerosis, ARRIVE - Use of Aspirin to Reduce Risk of Initial Vascular Events in patients at moderate risk of cardiovascular disease, ASCEND - A Study of Cardiovascular Events iN Diabetes, ASPREE - , BDT – British Doctor’s Trial, CI – confidence interval, ETDRS - Early Treatment Diabetic Retinopathy Study, HOT - Hypertension Optimal Treatment, JPAD - Japanese Primary prevention of Atherosclerosis with aspirin for Diabetes, JPPP - Japanese Primary Prevention Project, MACE – major adverse cardiovascular events, Non-CVD – Non-cardiovascular, PHS - Physician’s Health Study, POPADAD - Prevention Of Progression of Arterial Disease And Diabetes, PPP - Primary Prevention Project, TPT - Thrombosis Prevention Trial, WHS - Women’s Health Study*
